# Novel urinary glycan profiling by lectin array serves as the biomarkers for predicting renal prognosis in patients with IgA nephropathy

**DOI:** 10.1038/s41598-020-77736-1

**Published:** 2021-02-09

**Authors:** Chieko Kawakita, Koki Mise, Yasuhiro Onishi, Hitoshi Sugiyama, Michihiro Yoshida, Masao Yamada, Jun Wada

**Affiliations:** 1 Department of Nephrology, Rheumatology, Endocrinology and Metabolism, Okayama University Graduate School of Medicine, Dentistry and Pharmaceutical Sciences, 2-5-1 Shikata-cho, Kita-ku, Okayama, 700-8558 Japan; 2 Department of Human Resource Development of Dialysis Therapy for Kidney Disease, Okayama University Graduate School of Medicine, Dentistry and Pharmaceutical Sciences, Okayama, Japan; 3 Center for Innovative Clinical Medicine, Okayama University Hospital, Okayama, Japan; 4 GlycoTechnica Ltd, Yokohama, Japan

**Keywords:** Biochemistry, Biomarkers, Nephrology

## Abstract

In IgA nephropathy (IgAN), IgA1 molecules are characterized by galactose deficiency in *O*-glycans. Here, we investigated the association between urinary glycosylation profile measured by 45 lectins at baseline and renal prognosis in 142 patients with IgAN. The primary outcome was estimated glomerular filtration rate (eGFR) decline (> 4 mL/min/1.73 m^2^/year), or eGFR ≥ 30% decline from baseline, or initiation of renal replacement therapies within 3 years. During follow-up (3.4 years, median), 26 patients reached the renal outcome (Group P), while 116 patients were with good renal outcome (Group G). Multivariate logistic regression analyses revealed that lectin binding signals of *Erythrina cristagalli* lectin (ECA) (odds ratio [OR] 2.84, 95% confidence interval [CI] 1.11–7.28) and *Narcissus pseudonarcissus* lectin (NPA) (OR 2.32, 95% CI 1.11–4.85) adjusted by age, sex, eGFR, and urinary protein were significantly associated with the outcome, and they recognize Gal(β1-4)GlcNAc and high-mannose including Man(α1-6)Man, respectively. The addition of two lectin-binding glycan signals to the interstitial fibrosis/tubular atrophy score further improved the model fitness (Akaike’s information criterion) and incremental predictive abilities (c-index, net reclassification improvement, and integrated discrimination improvement). Urinary *N*-glycan profiling by lectin array is useful in the prediction of IgAN prognosis, since ECA and NPA recognize the intermediate glycans during *N*-glycosylation of various glycoproteins.

## Introduction

Immunoglobulin A nephropathy (IgAN) is the most prevalent primary glomerulonephritis worldwide^1,2^. In a systematic review of 1,619 publications, IgAN is more frequent in Asia (45 per million/year in Japan) than in Caucasians (31 cases per million/year in France)^3^. The most cases with IgAN demonstrate chanced microhematuria and proteinuria, and they are characterized by the IgA dominant immune complex deposition in glomerular mesangial area, recognized only by renal biopsy. The clinical manifestation and course were varied; asymptomatic microhematuria, acute glomerulonephritis, rapidly progressive glomerulonephritis, chronic nephritis, nephrotic syndrome, and end-stage kidney disease (ESKD). The Oxford Classification of IgAN is the most widely accepted system for evaluating histological features in biopsy specimens with a minimum of 8 glomeruli. The MEST-C score, including mesangial hypercellularity (M), endocapillary cellularity (E), segmental sclerosis (S), interstitial fibrosis/tubular atrophy (T), and crescents (C) lesions, is predictive of clinical renal outcome. The supportive therapy, *i.e.* tight blood pressure control with renin-angiotensin aldosterone system (RAS) inhibitors, treatment of dyslipidemia with statins, and life-style modification, delayed the decline of renal function. The additional benefits were observed in the immunosuppressive therapy with corticosteroids in recent randomized clinical trial^4^. Although the efforts were made in diagnostic and therapeutic approaches, 20%-40% of IgAN patients progress to ESKD by 20 years after initial renal biopsy^5,6^. Although several predictors of the renal prognosis at the diagnosis have been recognized, including the estimated glomerular filtration rate (eGFR), proteinuria, hypertension and pathological grading, they are not specific for IgAN^7–9^. Therefore, unmet medical needs in the management of IgAN are the identification of reliable biomarkers to predict the renal prognosis at an early stage.

In the 2 human subclasses of IgA, IgA1 and IgA2, IgA1 is selectively deposited in the glomeruli and the human IgA1 contains clustered *O*-glycans in its hinge region. In normal conditions, N-acetylgalactosamine (GalNAc) is linked to a serine or threonine and galactose linked in β1,3 to GalNAc, while sialic acid has an α2,6 linkage with GalNAc and α2,3 linkage with galactose^10^. In this decade, the evidences involving galactose-deficient IgA1 (Gd-IgA1) on the pathogenesis of IgAN has emerged^11–13^. The down-regulation of β1,3-galactosyltransferase (β3GalT)^14^ and up-regulation of α-2,6 sialyltransferase gene^15^ may contribute the production of sialylation of galacotose-deficient IgA1, the key autoantigen in IgAN. Suzuki et al. fist reported that urinary Gd-IgA1 might be a disease-specific biomarker of IgAN^16^. Later, Berthoux et al. demonstrated that serum levels of Gd-IgA1-targeting IgG and/or IgA autoantibodies could be related to the progression of IgAN^17^.

We previously reported that urinary excretion of *N*- or *O*-linked glycans conjugated to core proteins quantified by a lectin array were able to predict the renal prognosis in patients with diabetes and the urinary glycan profile might well-reflect kidney specific glycosylation changes, that is not the case with serum glycan profile^18^. However, the association between the urinary glycan profiles and the renal prognosis of IgAN has not been investigated to the best of our knowledge. By lectin array, the overall changes in sugar chains on global glycoproteins can be quantified in an unbiased fashion. Herein, we studied the relationship between the urinary glycan profiling using 45 lectins and the renal prognosis in patients with biopsy-proven IgAN.

## Results

### Patient characteristics

Among 157 patients diagnosed with isolated IgAN who received a renal biopsy from December 2010 to August 2017 at Okayama University Hospital, 142 patients were enrolled in the current study (Supplementary Fig. 1). The baseline characteristics of the patients at the time of the renal biopsy are shown in Table 1. The patients were 42.7 ± 16.3 years old, and 48% men. The mean baseline eGFR was 70.6 ± 25.9 mL/min/1.73 m^2^, and the median 24-h urinary protein (UP) was 0.73 g/day (interquartile range [IQR] 0.27–1.53). The primary outcome was defined as an estimated glomerular filtration rate (eGFR) decline (> 4 mL/min/1.73 m^2^/year), or eGFR ≥ 30% decline from baseline, or initiation of renal replacement therapies within 3 years. During a median follow-up period of 3.4 years, 26 patients reached the renal outcome (Group P), while 116 patients were with good renal outcome (Group G). The systolic blood pressure (SBP), serum IgA levels, and UP were significantly higher in Group P (n = 26) than Group G (n = 116). The percentage of treatments, such as tonsillectomy and/or steroid therapy, was not significantly different between Group P and G. In addition, there was no significant difference in the use of antihypertensive agents, such as angiotensin converting enzyme inhibitor (ACE-I) or angiotensin receptor blocker (ARB) and calcium channel blocker, between two groups. At the final follow-up, UP was significantly reduced, and the patients treated with ACE-I or ARB was increased compared with baseline in both Group P and G (Supplementary Table 1). Furthermore, SBP, diastolic blood pressure (DBP) and mean arterial pressure (MAP) demonstrated no significant differences between 2 groups at the end of observation (Supplementary Table 2).

**Table 1:** Clinical characteristics at baseline.

Clinical parameters	All patients (n = 142)	Renal outcome		*P* value
		Good (G) (n = 116)	Poor (P) (n = 26)	
Male^b^	68 (48)	53 (46)	15 (58)	0.27
Age (years)^a^	42.7 ± 16.3	41.5 ± 15.0	48.2 ± 20.6	0.06
BMI (kg/m^2^)^a^	22.2 ± 3.2	22.3 ± 3.3	22.2 ± 3.0	0.88
SBP (mmHg)^a^	124.1 ± 17.7	122.1 ± 17.1	133.0 ± 18.2	< 0.01
DBP (mmHg)^a^	77.9 ± 12.2	77.9 ± 12.3	77.9 ± 12.3	0.99
MAP (mmHg)^a^	93.3 ± 13.0	92.6 ± 13.1	96.2 ± 12.2	0.20
Hypertension^b^	47 (33)	35 (30)	12 (46)	0.12
sCr (mg/dL)^a^	0.94 ± 0.37	0.92 ± 0.32	1.04 ± 0.55	0.14
eGFR (mL/min/1.73 m^2^)^a^	70.6 ± 25.9	70.3 ± 23.9	72.2 ± 34.1	0.74
IgA (mg/gL)^a^	310.7 ± 117.4	301.2 ± 108.4	352.9 ± 146.4	0.04
C3 (mg/dL)^a^	100.4 ± 18.0	99.7 ± 17.7	103.5 ± 19.5	0.33
IgA/C3^a^	3.2 ± 1.2	3.1 ± 1.1	3.5 ± 1.3	0.15
UP (g/day)^a^	0.73 (0.27–1.53)	0.69 (0.26–1.27)	0.90 (0.37–3.09)	0.04
Urine occult blood^c^	124 (87)	101 (87)	23 (88)	0.57
Smoke (never/past/current)^de^	81 (62)/33 (25)/16 (12)	66 (63)/26 (25)/13 (12)	15 (60)/7 (28)/3 (12)	0.83
Re-biopsy^c^	25 (18)	19 (16)	6 (23)	0.40
ACE-I or ARB^b^	40 (28)	32 (28)	8 (31)	0.74
Calcium channel blocker^b^	30 (21)	22 (19)	8 (31)	0.18
Tonsillectomy^b^	86 (61)	74 (64)	12 (46)	0.09
Steroid therapy^b^	101 (71)	84 (72)	17 (65)	0.48
TSP^b^	83 (58)	71 (61)	12 (46)	0.16

Data are mean ± standard deviation, n (%), or median (inter-quartile range) unless otherwise indicated.

BMI, body mass index; SBP, systolic blood pressure; DBP, diastolic blood pressure; MAP, mean arterial pressure; sCr, serum creatinine; eGFR, estimated glomerular filtration rate; UP, urinary protein; ACE-I, angiotensin converting enzyme inhibitor; ARB, angiotensin receptor blocker; TSP, tonsillectomy combined with steroid pulse therapy.

^a^Student’s *t*-test.

^b^Pearson’s chi-square test.

^c^Fisher’s exact test.

^d^Wilcoxon test.

^e^n = 130.

**Figure 1:**
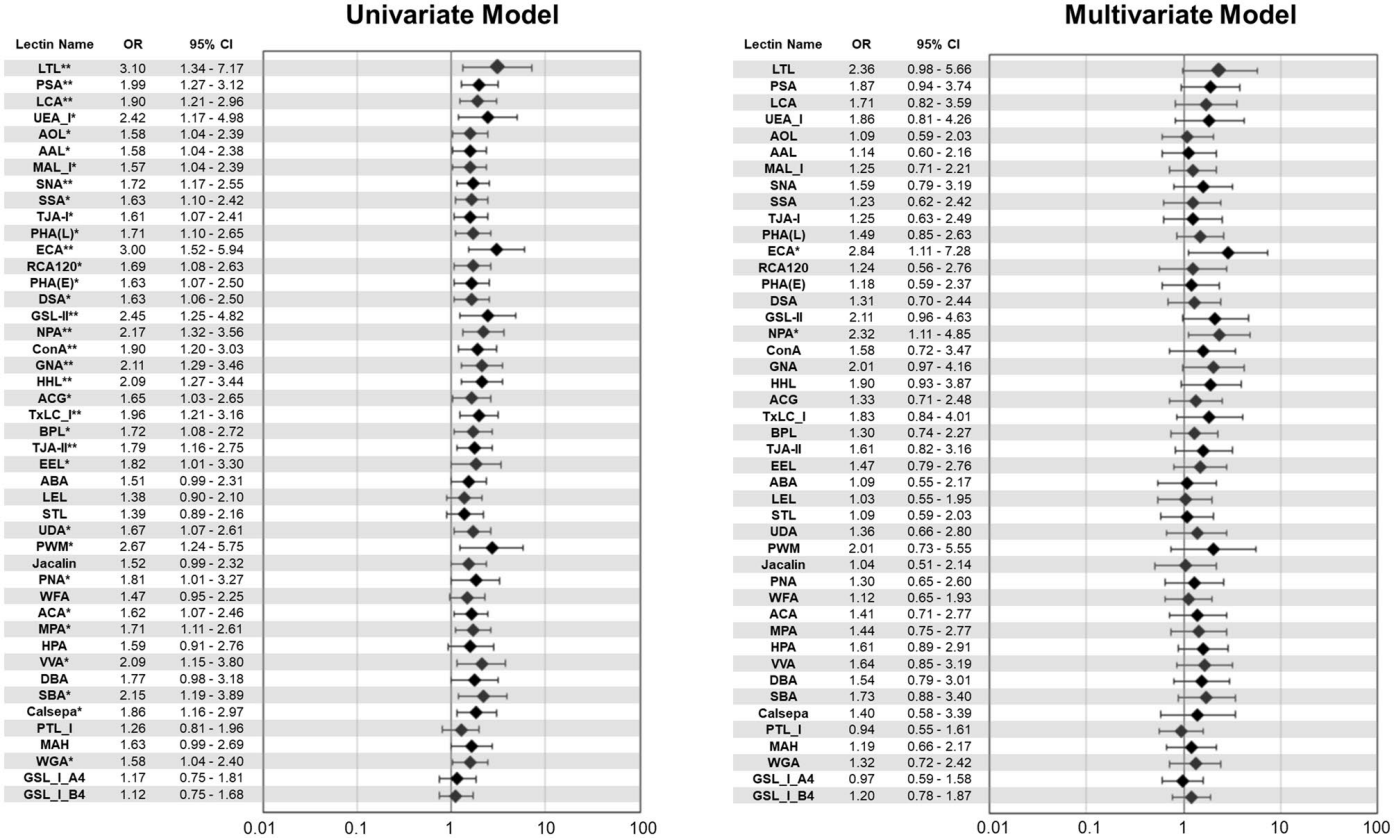
Univariate and multivariate logistic regression models for the renal outcome predicted by glycan indexes of 45 lectins. In the multivariate model, the OR for 1 SD of the net glycan intensity was adjusted for age, sex, eGFR, and log-transformed 24-h urinary protein at baseline. *P < 0.05, **P < 0.01. Abbreviations: OR, odds ratio; 95% CI, 95% confidence interval; eGFR, estimated glomerular filtration rate. (StataCorp. 2015. Stata Statistical Software: Release 14. College Station, TX: StataCorp LP)

**Table 2:** Univariate and multivariate logistic regression models using ECA signal and established risk factors to predict renal outcome.

	Univariate		Multivariate									
			Stepwise model		Model 1		Model 2		Model 3		Model 4	
	OR	P	OR	P	OR	P	OR	P	OR	P	OR	P
ECA (Net glycan intensity) (1 SD increments)	3.00	0.0016	3.48	0.001	2.84	0.03	2.69	0.04	2.66	0.04	2.67	0.05
Age (years)	1.03	0.06	1.05	0.01	1.05	0.01	1.05	0.02	1.05	0.03	1.06	0.01
Sex	1.62	0.27			1.72	0.27	1.63	0.33	1.57	0.37	1.90	0.21
eGFR (mL/min/1.73 m^2^)	1.00	0.74	1.03	0.01	1.04	0.01	1.03	0.01	1.04	0.01	1.07	0.0003
UP (g/day)	1.62	0.02			1.23	0.48	1.26	0.43	1.16	0.63	1.13	0.69
IgA (mg/dL)	1.00	0.05					1.00	0.37				
SBP (mmHg)	1.04	0.01							1.02	0.19		
**T score**												
T1	1.62	0.35									5.53	0.03
T2	5.00	0.02									22.77	0.01

Independent variables are as follow; Stepwise Model: ECA + Age + Sex + eGFR + UP; Model 1: ECA + Age + Sex + eGFR + UP; Model 2: ECA + Age + Sex + eGFR + UP + IgA; Model 3: ECA + Age + Sex + eGFR + UP + SBP; Model 4: ECA + Age + Sex + eGFR + UP + T score.

ECA, *Erythrina cristagalli* lectin; eGFR, estimated glomerular filtration rate; UP, urinary protein; SBP, systolic blood pressure; SD, standard deviation; OR, Odds ratio; P, *P*-value.

### Relationship between the renal outcome and lectin binding signals

The median follow-up period was 3.4 years (IQR 2.2–5.2 years). The data of net glycan intensity (Net-I) in Group P and G at baseline are shown in Supplementary Fig. 2. The lectin signals were generally higher in Group P versus Group G and the urinary protein excretions were higher in Group P versus Group G (Table 1). Cy3 fluorescent is labelled to amine-containing proteins and the background signals of albumin lacking glycosylation are the critical concerns regards the specificity of lectin array. Actually, Net-I in each lectin demonstrated significant correlation with urinary protein concentrations (Supplementary Fig. 3). However, the correlation matrix among 45 lectin binding signals demonstrated that r values between lectins with similar glycan recognition are very high, while r values between lectins with distinct glycan specificity are very low or even minus (Supplementary Fig. 4). These data supported the elimination of artifacts and specificity of urinary lectin array.

The odds ratios (ORs) for a poor renal outcome by 45 lectin binding signals from urine samples are shown in Fig. 1, and the reported glycan structures specific to each lectin are shown in Supplementary Table 3. Among 45 lectins, 34 lectin binding signals were significantly associated with the renal outcome in the univariate logistic regression model, whereas only two lectins (*Erythrina cristagalli* lectin [ECA] and *Narcissus pseudonarcissus* lectin [NPA]) were significantly related to the renal outcome in both univariate and multivariate models. In univariate models, the OR for the poor renal outcome by urinary ECA signals was 3.00 (95% confidence interval [CI], 1.52–5.94; p = 0.0016; false discovery rate [FDR] = 0.072 < 0.1) and by NPA 2.17 (95% CI, 1.32–3.56; p = 0.0021; FDR = 0.047 < 0.1). In the multivariate models adjusted for age, sex, eGFR, and log-transformed UP, the OR for the poor renal outcome by urinary ECA signals was 2.84 (95% CI, 1.11–7.28) and by NPA 2.32 (95% CI, 1.11–4.85) (Fig. 1). *Hippeastrum hybrid* lectin (HHL), *Canavalia ensiformis* lectin (ConA) and *Galanthus nivalis* lectin (GNA) demonstrated similar sugar recognition with NPA signals, while *Ricinus communis* lectin (RCA120) with ECA. In univariate analysis, these lectin binding signals demonstrate similar trends in Odds ratios and p values. However, a multivariate logistic regression analysis showed that the ECA and NPA remained statistically significant even after adjusting for established risk factors for progression of IgAN, *i.e.* age, sex, eGFR, and UP (Tables 2, 3). The inclusion of variables, *i.e.* IgA, SBP or T score into the models, ECA and NPA remained statistically significant. In the stepwise models, age, eGFR, and ECA/NPA were selected as statistically significant independent variables (Tables 2, 3). In another multivariate logistic regression models, we employed statistically significant parameters in univariate analyses, such as ECA/NPA, age, UP, IgA, SBP and T score, none of the parameters demonstrated significant contribution to the poor renal outcome. However, only ECA and NPA were selected as significant variables in stepwise models (Supplementary Tables 4 and 5). As shown in Supplementary Table 3, the glycans, Gal(β1-4)GlcNAc (Gal: galactose, GlcNAc: N-acetylglucosamine) and high-mannose (Man) including Man(α1-6)Man, were reported to bind to ECA and NPA, respectively. Since the glycosylation composition of IgG changes by biological age^19^ and 24 lectin signals among 45 lectins significantly correlated with age (Supplementary Fig. 5), we selected the patients with 19–64 years old both in Group P and G (40.4 ± 16.6, n = 20 *vs* 41.6 ± 12.7, n = 101) (P = 0.70), respectively. ECA (P = 0.047) but not NPA (P = 0.137) remained statistically significant even after adjusting for established risk factors for progression of IgAN, *i.e.* age, sex, eGFR, and UP in multivariate logistic regression analysis.

**Table 3:** Univariate and multivariate logistic regression models using NPA signal and established risk factors to predict renal outcome.

	Univariate		Multivariate									
			Stepwise model		Model 1		Model 2		Model 3		Model 4	
	OR	P	OR	P	OR	P	OR	P	OR	P	OR	P
NPA (Net glycan intensity) (1 SD increments)	2.17	0.01	2.65	0.001	2.32	0.02	2.21	0.04	2.13	0.04	2.11	0.05
Age (years)	1.03	0.06	1.05	0.005	1.06	0.0043	1.05	0.02	1.05	0.02	1.06	0.01
Sex	1.62	0.27			1.71	0.27	1.62	0.33	1.56	0.38	2.01	0.18
eGFR (mL/min/1.73 m^2^)	1.00	0.74	1.04	0.004	1.04	0.0026	1.04	0.01	1.04	0.0027	1.07	0.0001
UP (g/day)	1.62	0.02			1.18	0.58	1.22	0.51	1.14	0.66	1.10	0.76
IgA (mg/dL)	1.00	0.05					1.00	0.42				
SBP (mmHg)	1.04	0.01							1.02	0.23		
**T score**												
T1	1.62	0.35									5.25	0.04
T2	5.00	0.02									22.36	0.01

Independent variables are as follow; Stepwise Model: NPA + Age + Sex + eGFR + UP; Model 1: NPA + Age + Sex + eGFR + UP; Model 2: NPA + Age + Sex + eGFR + UP + IgA; Model 3: NPA + Age + Sex + eGFR + UP + SBP; Model 4: NPA + Age + Sex + eGFR + UP + T score.

NPA, *Narcissus pseudonarcissus* lectin; eGFR, estimated glomerular filtration rate; UP, urinary protein; SBP, systolic blood pressure; OR, Odds ratio; P, *P*-value.

**Table 4:** Pathological features.

Pathological parameters	All patients (n = 142)	Renal outcome		*P* value
		Good (G) (n = 116)	Poor (P) (n = 26)	
**Oxford classification**				
M1^b^	5 (4)	5 (4)	0 (0)	0.59
E1^a^	42 (30)	34 (29)	8 (31)	0.88
S1^a^	57 (40)	45 (39)	12 (46)	0.49
T^c^				
0	98 (69)	84 (72)	14 (54)	0.04
1	33 (23)	26 (22)	7 (27)	
2	11 (8)	6 (5)	5 (19)	
Cellular crescent^a^	29 (20)	24 (21)	5 (19)	0.87
Fibrocellular crescent^a^	68 (48)	57 (49)	11 (42)	0.53
Fibrous crescent^a^	30 (21)	24 (21)	6 (23)	0.79
Adhesion^a^	113 (80)	92 (79)	21 (81)	0.87
Percentage of global sclerosis^c^	10 (0–22)	10 (0–22)	10 (0–39)	0.83
Percentage of IFTA^c^	20 (10–0)	20 (10–30)	20 (10–50)	0.31
Glomerular number obtained by renal biopsy^c^	17 (12–21)	16 (12–21)	18 (12–24)	0.52

Data are n (%), or median (inter-quartile range) unless otherwise indicated.

Oxford classification; M1, Mesangial hypercellularity score > 0.5; E1, any endocapillary hypercellularity; S1, any segmental sclerosis; T, tubular atrophy and interstitial fibrosis (T0 ≤ 25%, 25% < T1 ≤ 50%, T2 > 50% of cortical area); IFTA, interstitial fibrosis/tubular atrophy.

^a^Pearson’s chi-square test.

^b^Fisher’s exact test.

^c^Wilcoxon test.

**Table 5:** Logistic regression analysis of the renal outcome.

Univariate model				Multivariate model			
Pathological parameters	Odds ratio	95%CI	*P* value	Pathological parameters	Odds ratio	95%CI	*P* value
**Oxford classification**				**Oxford classification**			
M1	–	–	–	M1	–	–	–
E1(vs. E0)	1.07	0.43–2.70	0.88	E1(vs. E0)	0.74	0.27–2.06	0.56
S1(vs. S0)	1.35	0.57–3.19	0.49	S1(vs. S0)	1.02	0.38–2.71	0.97
T1(vs. T0)	1.62	0.59–4.43	0.35	T1(vs. T0)	4.49	1.03–19.53	0.05
T2(vs. T0)	5.00	1.34–18.62	0.02	T2(vs. T0)	26.79	3.22–222.61	< 0.01
Cellular crescent^a^	0.91	0.31–2.67	0.87	Cellular crescent^a^	0.47	0.13–1.65	0.24
Fibrocellular crescent^a^	0.76	0.32–1.79	0.53	Fibrocellular crescent^a^	0.78	0.31–1.97	0.60
Fibrous crescent^a^	1.15	0.42–3.18	0.79	Fibrous crescent^a^	1.29	0.42–3.96	0.66
Adhesion^a^	1.10	0.37–3.21	0.87	Adhesion^a^	1.41	0.41–4.92	0.59
Global sclerosis^a^	1.01	0.99–1.04	0.20	Global sclerosis^a^	1.02	0.99–1.05	0.26
IFTA (10% increase)	1.26	1.02–1.55	0.04	IFTA (10% increase)	1.60	1.11–2.31	0.01

Oxford classification; M1, Mesangial hypercellularity score > 0.5; E1, any endocapillary hypercellularity; S1, any segmental sclerosis; T, tubular atrophy and interstitial fibrosis (T0 ≤ 25%, 25% < T1 ≤ 50%, T2 > 50% of cortical area).

CI, confidence interval; IFTA, interstitial fibrosis / tubular atrophy.

^a^The absence of each pathological parameter is defined as a reference.

**Figure 2:**
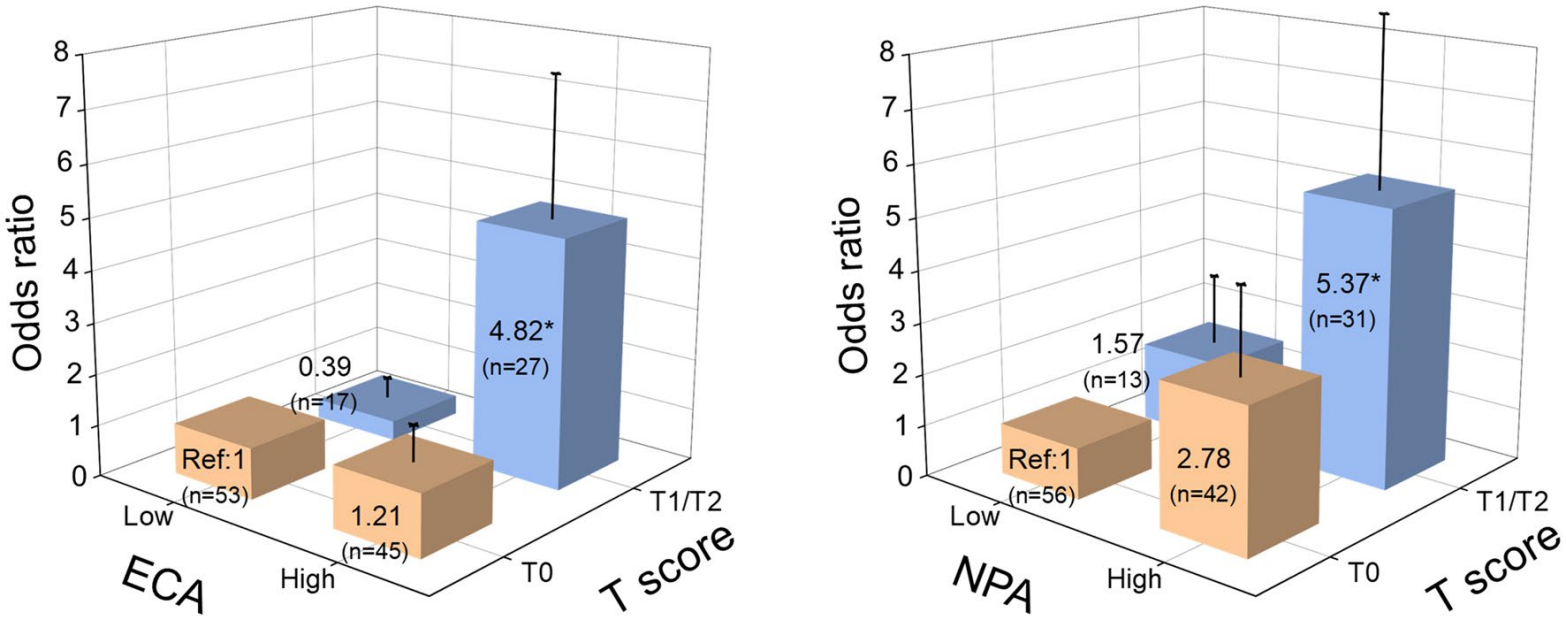
The comparisons of the odds ratios in the groups stratified according to ECA/NPA signals and T score. All patients were divided into four groups by lower or higher median lectin binding signals for ECA, NPA and T score (T0, T1, and T2). The odds ratio for renal outcome was calculated by a logistic regression analysis. The box and neighboring number indicate the odds ratio, and the bar shows the standard error. *P < 0.05 (vs reference group). Abbreviations: ECA, *Erythrina cristagalli* lectin; NPA, *Narcissus pseudonarcissus* lectin; Ref, reference. (StataCorp. 2015. Stata Statistical Software: Release 14. College Station, TX: StataCorp LP)

### Influence of pathological scoring, lectin binding signals and steroid use on the renal outcome

The Oxford classification and pathological grading are shown in Table 4. There were no statistically significant differences in most of the pathological findings between Group P and G, whereas T score of Oxford classification was significantly higher in Group P than in Group G (P = 0.04). The classification of pathological features and their renal outcomes are shown in Table 5. Although only 11 patients were in T2 category, T2 score of Oxford classification and percentage of interstitial fibrosis/tubular atrophy (IFTA) were significantly related to the renal outcome in both univariate and multivariate models. Next, we investigated the correlation of ECA and NPA with pathological parameters. ECA signals demonstrated mild correlation with T score (r = 0.25, P < 0.01), cellular crescent (r = 0.22, P = 0.01), global sclerosis (r = 0.22, P = 0.01), and IFTA (r = 0.21, P = 0.01), while NPA signals also revealed mild correlation with the T score (r = 0.32, P < 0.01), cellular crescent (r = 0.36, P < 0.01), and IFTA (r = 0.27, P < 0.01) (Supplementary Table 6). The comparisons of ORs in the groups stratified according to ECA/NPA signals and T score are shown in Fig. 2. In ECA_high_ and NPA_high_ 2-quantile groups, the elevation of risks for poor renal outcome were prominent in patients with severe interstitial disease (T1/T2). We further analyzed the association between steroid therapy and the renal outcome in subgroups stratified by MEST-C scores, including mesangial hypercellularity (M), segmental sclerosis (S), interstitial fibrosis/tubular atrophy (T) lesions, and crescents (C) (Supplementary Table 7). As a result, the patients with cellular crescent and adhesion were more likely to receive steroid therapy than those without cellular crescent (p = 0.01) or adhesion (p = 0.03). In contrast, the patients with higher T scores were more likely to avoid steroid therapy (p < 0.01).

**Table 6:** AIC, category-free NRI, and IDI for predicting the 3-year outcome with glycan index data, and difference of C-index between estimation models with or without glycan index and T score.

Depesndent variables	AIC	C-index (95% CI)	Difference of C-index (95% CI)	*P* value	Category-free NRI (95% CI)	*P* value	IDI (95% CI)	*P* value
Only covariates (crude)	127.6	0.76 (0.65–0.87)						
With ECA (Galβ1-4GlcNAc)	123.9	0.79 (0.67–0.90)	0.03 (− 0.02 to 0.07)	0.21	0.56 (0.09–1.02)	0.02	0.05 (− 0.01 to 0.11)	0.12
With NPA (High-Man including Manα1-6Man)	124.2	0.77 (0.66–0.89)	0.01 (− 0.04 to 0.06)	0.63	0.51 ( 0.08–0.94)	0.02	0.05 (− 0.02 to 0.13)	0.19
With T score	121.5	0.80 (0.69–0.91)	0.04 (− 0.01 to 0.09)	0.09	0.67 (0.17–1.17)	0.009	0.09 (− 0.01 to 0.19)	0.08
Combination with ECA and NPA	124.8	NA^a^	NA^a^		NA^a^		NA^a^	
Combination with ECA and T score	119.1	0.81 (0.71–0.92)	0.05 (0.00–0.11)	0.045	0.69 (0.20–1.19)	0.006	0.14 (0.01–0.26)	0.03
Combination with NPA and T score	119.4	0.81 (0.70–0.93)	0.05 (− 0.00 to 0.10)	0.06	0.70 (0.14–1.27)	0.01	0.13 (0.01–0.25)	0.03

Covariates (crude) were age, sex, estimated glomerular filtration rate, and log-transformed urinary protein excretion at the time of renal biopsy.

AIC, Akaike's information criterion; NRI, net reclassification improvement; IDI, integrated discrimination improvement; 95% CI, 95% confidence interval; C-index, concordance index; T score, tubular atrophy and interstitial fibrosis (T0 ≤ 25%, 25% < T1 ≤ 50%, T2 > 50% of cortical area); ECA, *Erythrina cristagalli* lectin; NPA, *Narcissus pseudonarcissus* lectin; NA, Not applied.

^a^In combination with ECA and NPA model, the AIC was higher than the single models of ECA, NPA, and T score, and other statistical analyses were not performed.

### Incremental predictive power of urinary glycan levels of ECA and NPA, plus T score

The Akaike information criterion (AIC) for evaluating the model fitting, concordance index (C-index), category-free net reclassification improvement (NRI), and integrated discrimination improvement (IDI) for predicting the primary renal outcome at the median follow-up time (3 years) obtained by adding the ECA, NPA, T score, and their combinations are summarized in Table 6. Adding the ECA, NPA, or T score to the multivariate model displayed improved the models, as shown by a decreased AIC and increased NRI. However, the addition of single parameters did not improve other model fitting indexes such as the C-index and IDI. Next, we investigated the effects of various combination of T score, ECA, and NPA signals. The combination of 2 parameters improved all of the incremental predictive abilities, *i.e.* ECA and T score; AIC decreased from 127.6 to 119.1, difference of C-index: 0.05 (95% CI: 0.00–0.11), category-free NRI: 0.69 (0.20–1.19), and IDI: 0.14 (0.01–0.26), NPA and T score; AIC decreased from 127.6 to 119.4, difference of C-index: 0.05 (-0.00–0.10), category-free NRI: 0.70 (0.14–1.27), and IDI: 0.13 (0.01–0.25). Receiver operating curves (ROC) of estimation models with and without glycan index and T score are shown in Supplementary Fig. 6. The calculated sensitivity, specificity, and accuracy by Youden’s method are shown in Supplementary Table 8. The addition of ECA or NPA increased sensitivity and further inclusion of T score improved specificity.

## Discussion

Glycans play pivotal roles in various physiological and pathological processes such as development, inflammation, autoimmune, hormone action, cell adhesion, and cancer^20–22^. In the current investigation, we firstly demonstrated that urinary excretion of glycans originated from the *N*-glycosylation process was tightly associated with the renal prognosis of IgAN. We found that the urinary excreted levels of glycans binding to ECA and NPA were significantly higher in IgAN patients with a poor renal outcome (Group P). Gal(β1-4)GlcNAc bound to ECA is involved in the process of both *N*- and *O*-glycosylation, while high-mannose including Man(α1-6)Man bound to NPA is mainly involved in *N*-glycosylation. It was recognized that aberrant *N*-glycosylation of IgA was associated with the pathogenesis of mouse IgAN^23^. Similarly, the abnormalities of *O*-glycans as well as the *N*-glycans in the aberrant IgA1 in human IgAN were reported^24^. Iwanami et al. showed that most of the *N*-glycans of the aberrant IgA1 were neutral complex- and high mannose-type, and the high mannose-type glycan was abundant and exclusively observed in the aberrant IgA1^24^. Although all 45 lectins including ECA and NPA recognize specific sugar structures, any protein carriers with specific sugar structures could be detected by lectin array systems and lectin signals are not confined to specific protein carrier, such as IgA. In organelles, *N*-glycosylation process begins in the endoplasmic reticulum (ER), and the complex- and hybrid- type glycans are synthesized from the high mannose-type glycans by its trimming and subsequent glycan elongation through the Golgi^25^. More specifically, high-mannose including Man(α1-6)Man is synthesized in the ER, *Cis*-Golgi, and part of the *Medial*-Golgi, subsequently complex- and hybrid- type glycans are generated in another part of the *Medial*-Golgi and *Trans*-Golgi^25,26^. In the final step in the Golgi, sialyltransferase, which enables sialic acid to bind to Gal(β1-4)GlcNAc, also functions in the *Trans*-Golgi (Supplementary Fig. 7a)^26^. The knockout of genes encoding α1,2-mannosidase-I and N-acetylglucosaminyl-transferase-I in HEK293 cells resulted in removed hybrid- and complex-type *N*-glycans and only high mannose-type *N*-glycans among recombinant proteins^27^. In addition, we previously raised the possibilities that urinary glycan excretion could reflect kidney-specific alterations of glycosylation rather than circulating serum glycosylation changes^18^. Taken together, the increased urinary high-mannose including Man(α1-6)Man and Gal(β1-4)GlcNAc could reflect the glycosylation abnormality in the *Trans*-Golgi and *Medial*-Golgi of renal tissues, and those glycosylation abnormalities might be involved in the progression of IgA nephropathy (Supplementary Fig. 7b).

Since the Oxford classification was published, a number of studies have proved that the classification is useful for predicting the renal prognosis of IgAN. The relationship of the renal prognosis with mesangial and endocapillary hypercellularity is still controversial, while IFTA has been reported to be a strong predictor of the renal outcome in several studies^28–31^. In our study, IFTA was significantly associated with the renal prognosis independent of baseline proteinuria and eGFR, which was compatible with previous reports^28–31^. Intriguingly, IFTA demonstrated mild correlation with the ECA and NPA signals, suggesting that the glycans detected by ECA and NPA might be involved in the mechanism of IFTA progression (Table 6). Furthermore, the urinary ECA and NPA signals had incremental predictive abilities when they were added to the model containing IFTA (Table 6). The addition of T scores in multivariate logistic regression model 1 in Tables 2 and 3; Supplementary Tables 4 and 5 did not alter the ORs of ECA and NPA signals, respectively. Therefore, we speculate that ECA/NPA binding glycans and interstitial renal injuries shown by IFTA are independently associated with progression of IgAN.

As well as IFTA, glomerular crescents tightly associated with a poor renal outcome of IgAN, resulting in the new inclusion of cellular and/or fibrocellular crescent scoring in the updated Oxford classification^32,33^. In the current investigation, crescents and segmental sclerosis were not associated with the renal prognosis. We found that patients with cellular crescents were more likely to receive steroid therapy than those without cellular crescents, and steroid therapy was associated with a good renal prognosis in the cellular crescent ( +) group. Likewise, in the patients with segmental sclerosis, those with steroid therapy tended to have a better renal prognosis, although the difference did not reach statistical significance (Supplementary Table 7). Given these associations, the treatment strategies and their therapeutic effects might affect the prognosis, resulting in different consequences from previous studies.

The localization of glycans in kidney tissues has been investigated only in the limited studies. ECA has been reported to bind to the proximal, distal tubules in the cortex, and the loops of Henle in the inner medulla on human kidney tissues^34^. The reported localization of ECA-recognizing Gal(β1-4)GlcNAc may support the link between elevation of urinary ECA signals and IFTA. NPA is a member of a large family of monocot mannose-binding proteins. The preferred glycan structures differ among lectins belonging to the same family. For example, NPA binds to α1-6-linked mannosyl residues, while GNA has a higher affinity to α1-3-linked mannosyl residues^35^. Since the relationship between NPA and kidney diseases has not been investigated, further experimental research is needed.

One of the limitations was the observational study with relatively smaller number of the enrolled patients. For example, only 26 renal events were observed, and 11 patients were classified as T2 category. We could not completely negate the possibility that the treatments for IgAN might not be standardized and the potential confounders could not be fully adjusted in the analyses. However, we observed no statistical differences in major treatment factors, such as ACE-I or ARB use, steroid therapy, and blood pressure control between Group P and G (Supplementary Table 2). Moreover, our sensitivity analyses revealed that ECA and NPA signals were still significant in the multivariate logistic regression analysis even after adjustment for various potential confounders (Tables 2, 3; Supplementary Tables 4 and 5). Another limitation is that the lectin microarray system may not determine the complete glycan structure and unknown preferred glycans to lectin potentially result in some bias. However, less time-consuming and more cost effective than conventional methods such as mass spectrometry (MS) were the benefits of lectin microarray^20^.

In conclusion, we showed that urinary excretion of glycans binding to two lectins, ECA recognizing Gal(β1-4)GlcNAc, and NPA binding to high-mannose including Man(α1-6)Man, were significantly associated with a poor renal prognosis in patients with IgAN. Furthermore, the addition of one of the two lectin binding signals and the Oxford classification T score to known renal prognostic factors can significantly improve the prediction of renal outcome. We need the further research to prove the underling mechanisms why the ECA and NPA signals could increase in urine of IgAN progressors and the abnormalities of glycosylation, especially *N*-glycosylation which was commonly recognized by ECA and NPA, might be involved with the progression of IgAN.

## Methods

### Study design

The current study was conducted as a retrospective cohort study. Among 157 patients diagnosed as “isolated IgAN” by performing biopsies from December 2010 to August 2017 at Okayama University Hospital, 142 were eligible for the enrollment. The patients with ≤ 3 glomeruli on biopsy specimens, < 1 year of follow-up, < 3 repeated measurements of eGFR, and a baseline eGFR < 10 mL/min/1.73 m^2^ were excluded (Supplementary Fig. 1).

### Ethics statement

This study was conducted in accordance with the principles of the Declaration of Helsinki, and the protocol was approved by the ethics committee of Okayama University Hospital (authorization number: 1709–039). Written informed consents were obtained from all participants. For patients < 18 years old, informed consent was obtained from parents or legal guardian. The study is registered with the University Hospital Medical Information Network Clinical Trials Registry (UMIN000029336).

### Lectin microarray analysis

Urine samples collected and stored at renal biopsy were used to measure urinary glycan levels. All specimens were stored at -80 °C, and thawed once to perform this study. We previously described a novel technique of glycan profiling by the evanescent-field fluorescence-assisted lectin microarray. In brief, 20 μL of urine samples were labeled with 100 μg of Cy3 (GE Healthcare) and free Cy3 was removed by Zeba Desalt Spin Column (Pearce). We applied urinary Cy3-labeled glycoproteins on the wells of LecChip Ver1.0 plotted with 45 lectins. The lectin binding signals were measured with GlycoStation Reader 1200 with scanning condition of Cumulative = 4 times, Exposure time = 299msec, Camera Gains = 75, 85, 95, 105, 115, 125 (GlycoTechnica Ltd, Tsukuba). Since Cy3 binds to primary amine in principle, and urinary albumin and creatinine were labeled by Cy3, causing minimal background reactivity. The data were analyzed by software, GlycoStation Tools Pro Ver.1.5., and the glycan index, *i.e.* the net glycan intensity (Net-I = raw glycan intensity [Raw-I] – background intensity [BG-I]) was used to quantify the lectin biding signals. In previous study, we investigated the variations between spot urine and 24-h collected urine data using 3 parameters, Net-I, Net-I/urinary creatinine (UCr), and Raw-I/UCr. As a result, Net-I values of spot urine most exactly reflected the 24-h urine among 3 parameters^18^. The changes of glycosylation modification in urinary proteins is quantitatively demonstrated by Net-I values, since the BG-I mainly reflects Cy3-albumin and Cy3-creatine and the subtraction of BG-I normalizes the albumin and creatinine concentrations in each urine sample. We performed all analyses by using the glycan index appropriately transformed from the Net-I according to its distribution.

### Laboratory parameters

Hypertension was defined as the baseline BP ≥ 140/90 mmHg or use of antihypertensive agents. For patients ≥ 18 years old, the eGFR was estimated by the equation: eGFR (mL/min/1.73 m^2^) = 194 × (serum creatinine [sCr] [mg/dL])^−1.094^ × (age [years])^−0.287^ (× 0.739 if female)^36^. For patients < 18 years old, the eGFR was calculated using the equation reported by the Japanese Society for Pediatric Nephrology^37^. Occult blood in urine was defined as > 5 urinary red blood cells /high-powered field in multiple urinalysis before a renal biopsy.

### Diagnosis and Oxford classification

The diagnosis of IgAN performed by 3 nephrologists and a renal pathologist by confirming mesangial proliferative glomerulonephritis in light microscopy, mesangial IgA deposition by immunofluorescence, and electron-dense deposits in the mesangial area by electron microscopy^38^. The following pathological scoring systems were employed, including MEST scores by Oxford classification^39^, presence of crescent formation (cellular/fibrocellular/fibrous) & tuft adhesion, glomeruli with global sclerosis (%), and IFTA (10% increments). There was very good agreement among 3 nephrologists’ scores in the percentage of IFTA (weighted *κ* value^40^: 0.92). The Oxford classification excludes cases with fewer than 8 glomeruli from analysis^39^. In this investigation, 9 patients (7 ≥ glomeruli ≥ 4) were included and the Oxford classification was performed by the consensus of 3 nephrologists.

### Outcomes

The primary outcome was defined as meeting at least one of the following criteria: eGFR decline of > 4 ml/min/1.73 m^2^/year during follow-up, 30% decline in eGFR from the baseline within three years, and commencement of renal replacement therapy for end-stage renal disease within the same period. The participants who reached the renal outcome were defined Group P (poor renal outcome; n = 26) and the others as Group G (good renal outcome; n = 116). We selected the composite outcome because the absolute eGFR decline, but the percent eGFR decline, is not affected by the baseline eGFR, and the composite outcome including absolute and percent eGFR decline is employed in a recent biomarker study^41–43^.

### Statistical analysis

Continuous variables are summarized as mean ± standard deviation (SD) or the median with the interquartile range, as appropriate. Categorical variables are presented as number of patients and proportions (%). Student’s *t*-test for continuous data and of Wilcoxon test for ordinal scale data are applied for comparing between two groups. Comparing the main clinical parameters at baseline and final follow-up was evaluated using the paired *t*-tests, the Wilcoxon signed rank test, or the McNemar test. Categorical variables were compared using Pearson’s chi-square test and Fisher’s exact test. Non-normally distributed variables were subjected to log-transformation to improve normality before analysis. To evaluate inter-observer concordance, we calculated weighted κ statistics^40^. The logistic regression model was used to calculate the OR and 95% CI. To avoid the type I errors in null hypothesis testing when conducting multiple comparisons, FDR is calculated by Benjamini and Hochberg. In the multivariate model, ORs were adjusted for age, sex, eGFR, and log-transformed UP (g/day) at the time of renal biopsy. These covariates were selected according to biological plausibility and the findings of previous reports^44^. In addition, as sensitivity analyses, other potential covariates were incorporated into the multivariate models one by one. We tested for a formal interaction of each glycan index with eGFR or log-transformed UP in the multivariate regression models. Any glycan index × eGFR/log-transformed UP was not statistically significant. We also divided all patients into four groups by median of the glycan index (ECA or NPA) and T score (T0 / T1 or T2), and calculated the ORs to the renal outcome by a logistic regression analysis. Furthermore, several analyses were employed to evaluate the incremental predictive value of preferable glycan biomarkers and pathological scores. We first used AIC to compare the model fitting. Next, C-index was compared between multivariate logistic regression models with or without biomarkers. Finally, improvement in discriminating the three-year risk of the outcome was assessed by analysis of category-free NRI and IDI, as reported elsewhere^45–47^. The 95% CIs for the differences in the C-index, category-free NRI, and IDI were computed based on 500 bootstrap samples. ROC of estimation models with and without glycan index and T score were used to evaluate the characteristics of biomarkers. The cutoff points were calculated by Youden’s method. Two-tailed P < 0.05 was considered to indicate statistical significance. Analyses and creation of graphs were performed with StataCorp. 2015. Stata Statistical Software: Release 14. College Station, TX: StataCorp LP. and Origin version 2018 (OriginLab Corporation, MA, USA) software programs.

## Supplementary information

Supplementary Information 1.

Supplementary Information 2.

## Data Availability

The main clinical data and lectin binding signal data generated during the current study are available in the Supplementary datasheet.
