# Supplementary material for: Novel urinary glycan profiling by lectin array serves as the biomarkers for predicting renal prognosis in patients with IgA nephropathy
Source: Sci Rep. 2021 Feb 9;11:3394. doi: 10.1038/s41598-020-77736-1 (PMC7873239; doi:10.1038/s41598-020-77736-1)
Supplement: Supplementary file 2 — Supplementary Information 2. [file 41598_2020_77736_MOESM2_ESM.pdf]

Supplementary Information

Novel urinary glycan profiling by lectin array serves as the biomarkers for predicting renal prognosis in patients with IgA nephropathy

Chieko Kawakita, MD, Koki Mise, MD, PhD, Yasuhiro Onishi, MD, Hitoshi Sugiyama, MD, PhD, Michihiro Yoshida, PhD, Masao Yamada, PhD, and Jun Wada, MD, PhD

Supplementary Table 1. Comparison of main clinical parameters between baseline and final follow-up

Clinical parameters	All patients (n=142)			Renal outcome					
				Good (G)			Poor (P)		
	Baseline (n=142)	At final follow-up (n=126)	*P*-value	Baseline (n=116)	At final follow-up (n=103)	*P*-value	Baseline (n=26)	At final follow-up (n=23)	*P*-value
SBP (mmHg)*	124.1 ± 17.7	124.7 ± 15.6	0.55	122.1 ± 17.1	123.9 ± 15.4	0.15	133.0 ± 18.2	128.5 ± 16.4	0.27
DBP (mmHg)*	77.9 ± 12.2	75.5 ± 11.0	0.01	77.9 ± 12.3	75.6 ± 10.7	0.05	77.9 ± 12.3	74.7 ± 12.3	0.09
MAP (mmHg)*	93.3 ± 13.0	91.9 ± 11.6	0.19	92.6 ± 13.1	91.7 ± 11.5	0.55	96.2 ± 12.2	92.7 ± 12.3	0.12
sCr (mg/dL)*	0.94 ± 0.37	1.06 ± 0.56	<0.01	0.92 ± 0.32	0.93 ± 0.33	0.41	1.04 ± 0.55	1.60 ± 0.94	<0.01
eGFR* (mL/min/1.73m^2)	70.6 ± 25.9	64.6 ± 26.3	<0.01	70.3 ± 23.9	67.7 ± 24.2	0.02	72.2 ± 34.1	50.6 ± 31.1	<0.01
UP (g/day)†	0.73 (0.27-1.53)	0.22 (0.08-0.67)**	<0.01	0.69 (0.26-1.27)	0.21 (0.08-0.60)	<0.01	0.90 (0.37-3.09)	0.35 (0.12-1.38)	0.01
ACE-I or ARB‡	40 (28)	59 (47)	<0.01	32 (28)	46 (45)	<0.01	8 (31)	13 (57)	0.05
Calcium channel blocker‡	30 (21)	31 (25)	0.31	22 (19)	23 (22)	0.48	8 (31)	8 (35)	0.32

Data are mean ± standard deviation, n (%), or median (inter-quartile range). Abbreviations; SBP, systolic blood pressure; DBP, diastolic blood pressure; MAP, mean arterial pressure; sCr, serum creatinine; eGFR, estimated glomerular filtration rate; UP, urinary protein; ACE-I, angiotensin converting enzyme inhibitor; ARB, angiotensin receptor blocker. *Paired *t*-test, †Wilcoxon signed rank test, ‡McNemar test, ** n=119.

Supplementary Table 2. Comparison of main clinical parameters at final follow-up in patients with and without renal outcome

Clinical parameters	All patients (n=126)	Renal outcome		*P*-value
		Good (G) (n=103)	Poor (P) (n=23)	
SBP (mmHg)*	124.7 ± 15.6	123.9 ± 15.4	128.5 ± 16.4	0.20
DBP (mmHg)*	75.5 ± 11.0	75.6 ± 10.7	74.7 ± 12.3	0.72
MAP (mmHg)*	91.9 ± 11.6	91.7 ± 11.5	92.7 ± 12.3	0.73
sCr (mg/dL)*	1.06 ± 0.56	0.93 ± 0.33	1.60 ± 0.94	<0.01
eGFR (mL/min/1.73m^2)*	64.6 ± 26.3	67.7 ± 24.2	50.6 ± 31.1	<0.01
UP (g/day)‡**	0.22 (0.08-0.67)	0.21 (0.08-0.60)	0.35 (0.12-1.38)	0.06
ACE-I or ARB†	59 (47)	46 (45)	13 (57)	0.30
Calcium channel blocker†	31 (25)	23 (22)	8 (35)	0.21

Data are mean ± standard deviation, n (%), or median (inter-quartile range). Abbreviations; SBP, systolic blood pressure; DBP, diastolic blood pressure; MAP, mean arterial pressure; sCr, serum creatinine; eGFR, estimated glomerular filtration rate; UP, urinary protein; ACE-I, angiotensin converting enzyme inhibitor; ARB, angiotensin receptor blocker. *Student's *t*-test, †Pearson's chi-square test, ‡Wilcoxon test, **n=119.

Supplementary Table 3. Preferred glycan structures binding to 45 lectins with different specificity

Lectin Name	Origin	Lectin Family	Monosaccharide Specificity	Preferred glycan structure (terminal epitope)
**LTL**	*Lotus tetragonolobus*	Legume (L-type)	Fuc	Fuc(α1-3)(Gal(β1-4))GlcNAc (Lex), Fuc(α1-2)Gal(β1-4)GlcNAc (H-type 2)
**PSA**	*Pisum sativum*	Legume (L-type)	Fuc/Man	Fuc(α1-6)GlcNAc, High-Man
**LCA**	*Lens culinaris*	Legume (L-type)	Fuc/Man	Fuc(α1-6)GlcNAc, High -Man
**UEA_I**	*Ulex europaeus*	Legume (L-type)	Fuc	Fuc(α1-2)Gal(β1-4)GlcNAc (H-type 2)
**AOL**	fungus*, Aspergillus oryzae*	Fucose lectin	Fuc	Fuc(α1-6)GlcNAc (core Fuc), Fuc(α1-2)Gal(β1-4)GlcNAc (H-type 2)
**AAL**	*Aleuria aurantia*	Fucose lectin	Fuc	Fuc(α1-6)GlcNAc (core Fuc), Fuc(α1-3)(Gal(β1-4))GlcNAc (Lex)
**MAL_I**	*Maackia amurensis*	Legume (L-type)	Sia	Sia(α2-3)Gal(β1-4)GlcNAc
**SNA**	*Sambucus nigra*	Ricin B-cahin-like (R-type)	Sia	Sia(α2-6)Gal/GalNAc
**SSA**	*Sambucus sieboldiana*	Ricin B-cahin-like (R-type)	Sia	Sia(α2-6)Gal/GalNAc
**TJA-I**	*Trichosanthes japonica*	Ricin B-cahin-like (R-type)	Sia	Sia(α2-6)Gal/GalNAc
**PHA(L)**	*Phaseolus vulgaris*	Legume (L-type)	Complex	Tri/tetra-antennary complex-type *N*-glycan
**ECA**	*Erythrina cristagalli*	Legume (L-type)	Gal	Gal(β1-4)GlcNAc
**RCA120**	*Ricinus communis*	Ricin B-cahin-like (R-type)	Gal	Gal(β1-4)GlcNAc
**PHA(E)**	*Phaseolus vulgaris*	Legume (L-type)	Gal	*N*-glycans with outer Gal and bisecting GlcNAc
**DSA**	*Datura stramonium*	Hevein (Chitin-type)	GlcNAc	(GlcNAc(β1-4))n, triantennary, tetraantennary *N*-glycans

**GSL-II**	*Griffonia simplicifolia*	Legume (L-type)	GlcNAc	Agalactosylated tri/tetra antennary glycans, GlcNAc
**NPA**	*Narcissus pseudonarcissus*	Monocot (GNA-related)	Man	High-Man including Man(α1-6)Man
**ConA**	*Canavalia ensiformis*	Legume (L-type)	Man	High-Man including Man(α1-6)(Man(α1-3))Man
**GNA**	*Galanthus nivalis*	Monocot (GNA-related)	Man	High-Man including Man(α1-3)Man
**HHL**	*Hippeastrum hybrid*	Monocot (GNA-related)	Man	High-Man including Man(α1-3)Man or Man(α1-6)Man
**ACG**	*Agrocybe cylindracea*	Galectin	Gal	Sia(α2-3)Gal(β1-4)GlcNAc
**TxLC_I**	*Tulipa gesneriana*	Monocot (GNA-related)	Man/GalNAc	Man(α1-3)(Man(α1-6))Man, bi- and tri-antennary *N*-glycans, GalNAc
**BPL**	*Bauhinia purpurea alba*	Legume (L-type)	Gal	Gal(β1-3)GalNAc, GalNAc
**TJA-II**	*Trichosanthes japonica*	Others	Gal	Fuc(α1-2)Galβ1, GalNAcβ1
**EEL**	*Euonymus europaeus*	Legume (L-type)	Gal	Gal(α1-3)Gal(β1-4)GlcNAc, Fuc(α1-2)(Gal(α1-3))Gal(β1-4)GlcNAc
**ABA**	fungus, *Agaricus bisporus*	Others	Gal, GlcNAc	Gal(β1-3)GalNAc, GlcNAc
**LEL**	tomato, *Lycopersicon esculentum*	Hevein (Chitin-type)	GlcNAc	(GlcNAc(β1-4))n, (Gal(β1-4)GlcNAc)n (polylactosamine)
**STL**	potato, *Solanum tuberosum*	Hevein (Chitin-type)	GlcNAc	(GlcNAc(β1-4))n, (GlcNAc(β1-4)MurNAc)n (peptidoglycan backbone)
**UDA**	*Urtica dioica*	Hevein (Chitin-type)	GlcNAc	GlcNAc(β1-4)GlcNAc, Man5～Man9
**PWM**	pokeweed, *Phytolacca americana*	Hevein (Chitin-type)	GlcNAc	(GlcNAc(β1-4))n
**Jacalin**	*Artocarpus integrifolia*	Jacalin	Gal	Gal(β1-3)GalNAc, αGalNAc (6O-unsubstituted)
**PNA**	peanut, *Arachis hypogaea*	Legume (L-type)	Gal	Gal(β1-3)GalNAc

**WFA**	*Wisteria floribunda*	Legume (L-type)	GalNAc	GalNAc(β1-4)GlcNAc, Gal(β1-3(-6))GalNAc
**ACA**	*Amaranthus caudatus*	Ricin B-cahin-like (R-type)	Gal	Gal(β1-3)GalNAc
**MPA**	*Maclura pomifera*	Jacalin	Gal	Gal(β1-3)GalNAc, GalNAc
**HPA**	snail, *Helix pomatia agglutinin*	Discoidin	GalNAc	αGalNAc
**VVA**	*Vicia villosa*	Legume (L-type)	GalNAc	αGalNAc, GalNAc(α1-3)Gal
**DBA**	*Dolichos biflorus*	Legume (L-type)	GalNAc	Blood group A antigen, GalNAc(α1-3)GalNAc
**SBA**	soybean, *Glycine max*	Legume (L-type)	GalNAc	GalNAc, GalNAc(α1-3)Gal
**Calsepa**	*Calystegia sepium*	Jacalin	Man	High-Man (Man2–6), *N*-glycans including bisecting GlcNAc
**PTL_I**	*Psophocarpus tetragonolobus*	Legume (L-type)	GalNAc	αGalNAc
**MAH**	*Maackia amurensis*	Legume (L-type)	Sia	Sia(α2-3)Gal(β1-3)(Sia(α2-6))GalNAc
**WGA**	wheat germ, *Triticum unlgaris*	Hevein (Chitin-type)	GlcNAc	(GlcNAc(β1-4))n, NeuAc
**GSL_I_A4**	*Griffonia simplicifolia Lectin I Isolectin A4*	Legume (L-type)	GalNAc	αGalNAc
**GSL_I_B4**	*Griffonia simplicifolia Lectin I Isolectin B4*	Legume (L-type)	Gal	αGal

Abbreviations; Fuc, Fucose; Gal, Galactose; GlcNAc, N-acetylglucosamine ; Man, Mannose; Sia, Sialic acid; GalNAc, N-acetylgalactosamine.

Supplementary Table 4. Univariate and multivariate logistic regression models using ECA signal and independent variables with statistical significance to predict renal outcome

		Univariate		Multivariate			
				Stepwise Model		Model 1	
		OR	P	OR	P	OR	P
**ECA (Net glycan intensity) (1 SD increments)**		3.00	0.0016	3.00	0.002	2.28	0.07
**Age (years)**		1.03	0.06			1.00	1.00
**UP (g/day)**		1.62	0.02			0.95	0.85
**IgA (mg/dL)**		1.00	0.05			1.00	0.15
**SBP (mmHg)**		1.04	0.01			1.02	0.28
**T score**	**T1**	1.62	0.35			1.15	0.81
	**T2**	5.00	0.02			2.52	0.30

Independent variables are as follow; Stepwise Model: ECA + Age + UP + IgA + SBP + T score; Model 1: ECA + Age + UP + IgA + SBP + T score.

Abbreviations; ECA, *Erythrina cristagalli*; UP, urinary protein; SBP, systolic blood pressure; SD, standard deviation; OR, Odds ratio; P, *P*-value.

Supplementary Table 5. Univariate and multivariate logistic regression models using NPA signal and independent variables with statistical significance to predict renal outcome

		Univariate		Multivariate			
				Stepwise Model		Model 1	
		OR	P	OR	P	OR	P
**NPA (Net glycan intensity) (1 SD increments)**		2.17	0.01	2.17	0.002	1.67	0.16
**Age (years)**		1.03	0.06			1.00	0.92
**UP (g/day)**		1.62	0.02			0.98	0.95
**IgA (mg/dL)**		1.00	0.05			1.00	0.14
**SBP (mmHg)**		1.04	0.01			1.02	0.28
**T score**	**T1**	1.62	0.35			1.12	0.85
	**T2**	5.00	0.02			2.58	0.28

Independent variables are as follow; Stepwise Model: NPA + Age + UP + IgA + SBP + T score; Model 1: NPA + Age + UP + IgA + SBP + T score.

Abbreviations; NPA, *Narcissus pseudonarcissus*; UP, urinary protein; SBP, systolic blood pressure; SD, standard deviation; OR, Odds ratio; P, *P*-value.

Supplementary Table 6: Correlations between two lectin signals and pathological parameters in patients with IgA nephropathy

ECA			
Pathological parameter		*r*	*P*-value
Oxford classification	M	0.00	0.96
	E	0.14	0.09
	S	0.20	0.02
	T	0.25	<0.01
Cellular crescent		0.22	0.01
Fibrocellular crescent		-0.06	0.52
Fibrous crescent		-0.06	0.46
Adhesion		0.06	0.48
Global sclerosis		0.22	0.01
IFTA (10% increase)		0.21	0.01

NPA			
Pathological parameter		*r*	*P*-value
Oxford classification	M	0.00	1.00
	E	0.08	0.32
	S	0.18	0.03
	T	0.32	<0.01
Cellular crescent		0.36	<0.01
Fibrocellular crescent		-0.03	0.77
Fibrous crescent		-0.03	0.71
Adhesion		0.03	0.68
Global sclerosis		0.16	0.05
IFTA (10% increase)		0.27	<0.01

Abbreviations; ECA, *Erythrina cristagalli*; NPA, *Narcissus pseudonarcissus*; *r*, Spearman correlation coefficient; M, Mesangial hypercellularity score; E, endocapillary hypercellularity; S, segmental sclerosis; T, tubular atrophy and interstitial fibrosis; IFTA, interstitial fibrosis/tubular atrophy.

Supplementary Table 7. Association between steroid therapy and the outcome in the group stratified by pathological scoring

		M score‡					
		M0	Steroid therapy		M1	Steroid therapy	
			(-)	(+)		(-)	(+)
		137 (96%)	40 (28%)	97 (68%)	5 (4%)	1 (1%)	4 (3%)
Renal outcome	Good (G)	111 (78%)	31 (22%)	80 (56%)	5 (4%)	1 (1%)	4 (3%)
	Poor (P)	26 (18%)	9 (6%)	17 (12%)	0 (0%)	0 (0%)	0 (0%)

		E score†					
		E0	Steroid therapy		E1	Steroid therapy	
			(-)	(+)		(-)	(+)
		100 (70%)	31 (22%)	69 (49%)	42 (30%)	10 (7%)	32 (23%)
Renal outcome	Good (G)	82 (58%)	25 (18%)	57 (40%)	34 (24%)	7 (5%)	27 (19%)
	Poor (P)	18 (13%)	6 (4%)	12 (8%)	8 (6%)	3 (2%)	5 (4%)

		S score†					
		S0	Steroid therapy		S1	Steroid therapy	
			(-)	(+)		(-)	(+)
		85 (60%)	25 (18%)	60 (42%)	57 (40%)	16 (11%)	41 (29%)
Renal outcome	Good (G)	71 (50%)	21 (15%)	50 (35%)	45 (32%)	11 (8%)	34 (24%)
	Poor (P)	14 (10%)	4 (3%)	10 (7%)	12 (8%)	5 (4%)	7 (5%)

		T score§								
		**T0**	**Steroid therapy**		**T1**	**Steroid therapy**		**T2**	**Steroid therapy**	
			(-)	(+)		(-)	(+)		(-)	(+)
		98 (69%)	21 (15%)	77 (54%)	33 (23%)	14 (10%)	19 (13%)	11 (8%)	6 (4%)	5 (4%)*
**Renal outcome**	**Good (G)**	84 (59%)	20 (14%)	64 (45%)	26 (18%)	11 (8%)	15 (11%)	6 (4%)	1 (1%)	5 (4%)
	**Poor (P)**	14 (10%)	1 (1%)	13 (9%)	7 (5%)	3 (2%)	4 (3%)	5 (4%)	5 (4%)	0 (0%)

		**Cellular crescent†**					
		**(-)**	**Steroid therapy**		**(+)**	**Steroid therapy**	
			(-)	(+)		(-)	(+)
		113 (80%)	38 (27%)	75 (53%)	29 (20%)	3 (2%)	26 (18%)*
**Renal outcome**	**Good (G)**	92 (65%)	31 (22%)	61 (43%)	24 (17%)	1 (1%)	23 (16%)
	**Poor (P)**	21 (15%)	7 (5%)	14 (10%)	5 (4%)	2 (1%)	3 (2%)

		**Fibrocellular crescent†**					
		**(-)**	**Steroid therapy**		**(+)**	**Steroid therapy**	
			(-)	(+)		(-)	(+)
		74 (52%)	21 (15%)	53 (37%)	68 (48%)	20 (14%)	48 (34%)
**Renal outcome**	**Good (G)**	59 (42%)	15 (11%)	44 (31%)	57 (40%)	17 (12%)	40 (28%)
	**Poor (P)**	15 (11%)	6 (4%)	9 (6%)	11 (8%)	3 (2%)	8 (6%)

		Fibrous crescent†					
		(-)	Steroid therapy		(+)	Steroid therapy	
			(-)	(+)		(-)	(+)
		112 (79%)	30 (21%)	82 (58%)	30 (21%)	11 (8%)	19 (13%)
Renal outcome	Good (G)	92 (65%)	22 (15%)	70 (49%)	24 (17%)	10 (7%)	14 (10%)
	Poor (P)	20 (14%)	8 (6%)	12 (8%)	6 (4%)	1 (1%)	5 (4%)

		Adhesion†					
		(-)	Steroid therapy		(+)	Steroid therapy	
			(-)	(+)		(-)	(+)
		29 (20%)	13 (9%)	16 (11%)	113 (80%)	28 (20%)	85 (60%)*
Renal outcome	Good (G)	24 (17%)	10 (7%)	14 (10%)	92 (65%)	22 (15%)	70 (49%)
	Poor (P)	5 (4%)	3 (2%)	2 (1%)	21 (15%)	6 (4%)	15 (11%)

		Global sclerosis§								
		G0	Steroid therapy		G1	Steroid therapy		G2	Steroid therapy	
			(-)	(+)		(-)	(+)		(-)	(+)
		115 (81%)	30 (21%)	85 (60%)	22 (15%)	8 (6%)	14 (10%)	5 (4%)	3 (2%)	2 (1%)
Renal outcome	Good (G)	97 (68%)	27 (19%)	70 (49%)	17 (12%)	5 (4%)	12 (8%)	2 (1%)	0 (0%)	2 (1%)
	Poor (P)	18 (13%)	3 (2%)	15 (11%)	5 (4%)	3 (2%)	2 (1%)	3 (2%)	3 (2%)	0 (0%)

Oxford classification; M1, Mesangial hypercellularity score >0.5; E1, any endocapillary hypercellularity; S1, any segmental sclerosis; T, tubular atrophy and interstitial fibrosis (T0 ≤ 25%, 25% < T1 ≤ 50%, T2 > 50% of cortical area).

Global sclerosis (G) was classified into 3 groups according to the percentage of global sclerosis: G0 (≤25% of glomeruli), G1 (26–50% of glomeruli), and G2 (>50% of glomeruli).

* $P<0.05$ (vs. patients without each pathological lesion). [†]Pearson's chi-square test, [‡]Fisher's exact test, [§]Wilcoxon test.

Supplementary Table 8. Diagnostic ability between estimation models with or without glycan index and T score

Estimation model	Sensitivity (%)	Specificity (%)	Accuracy (%)	C-index (95% CI)	Difference of C-index (95%CI)	*P*-value
Only covariates (crude)	76.9	75.9	76.0	0.76 (0.65-0.87)		
With ECA (Galβ1-4GlcNAc)	80.8	74.1	75.4	0.79 (0.67-0.90)	0.03 (-0.02-0.07)	0.21
With NPA (High-Man including Manα1-6Man)	84.6	65.5	69.0	0.77 (0.66-0.89)	0.01 (-0.04-0.06)	0.63
With T score	76.9	81.9	81.0	0.80 (0.69-0.91)	0.04 (-0.01-0.09)	0.09
Combination with ECA and T score	69.2	85.3	82.4	0.81 (0.71-0.92)	0.05 (0.00-0.11)	0.045
Combination with NPA and T score	84.6	73.3	75.4	0.81 (0.70-0.93)	0.05 (-0.00-0.10)	0.06

Covariates (crude) were age, sex, estimated glomerular filtration rate, and log-transformed urinary protein excretion at the time of renal biopsy. Abbreviations; 95% CI, 95% confidence interval; C-index, concordance index; ECA, *Erythrina cristagalli*; NPA, *Narcissus pseudonarcissus*; T score, tubular atrophy and interstitial fibrosis (T0≤25%, 25%<T1≤50%, T2>50% of cortical area).
Youden's method was applied to determine the best cutoff point, and sensitivity, specificity, and accuracy were calculated at the best cutoff point.

Supplementary Figure 1

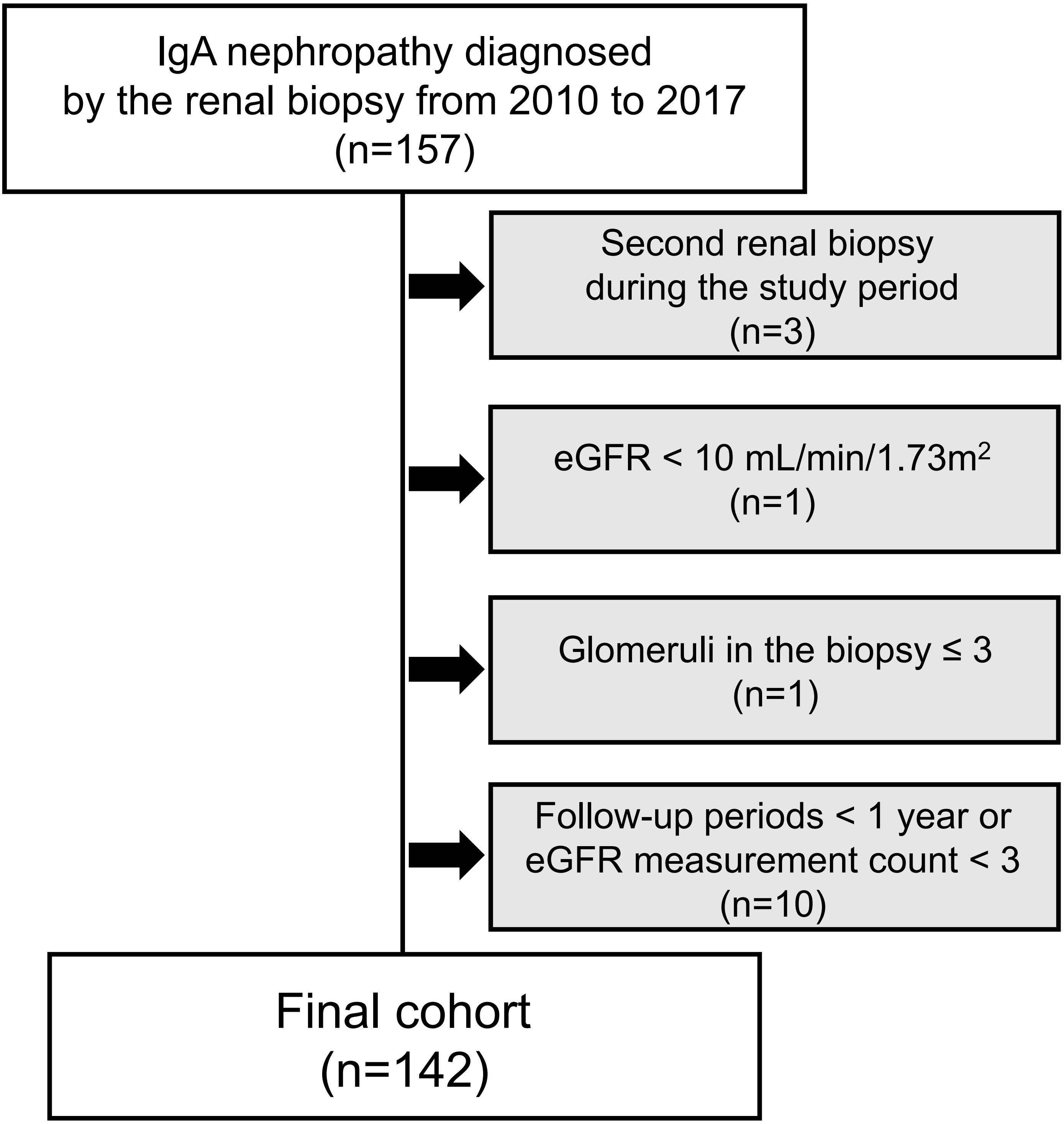

Supplementary Figure 1. Flow diagram of current clinical study. Abbreviations: eGFR, estimated glomerular filtration rate.

Supplementary Figure 2

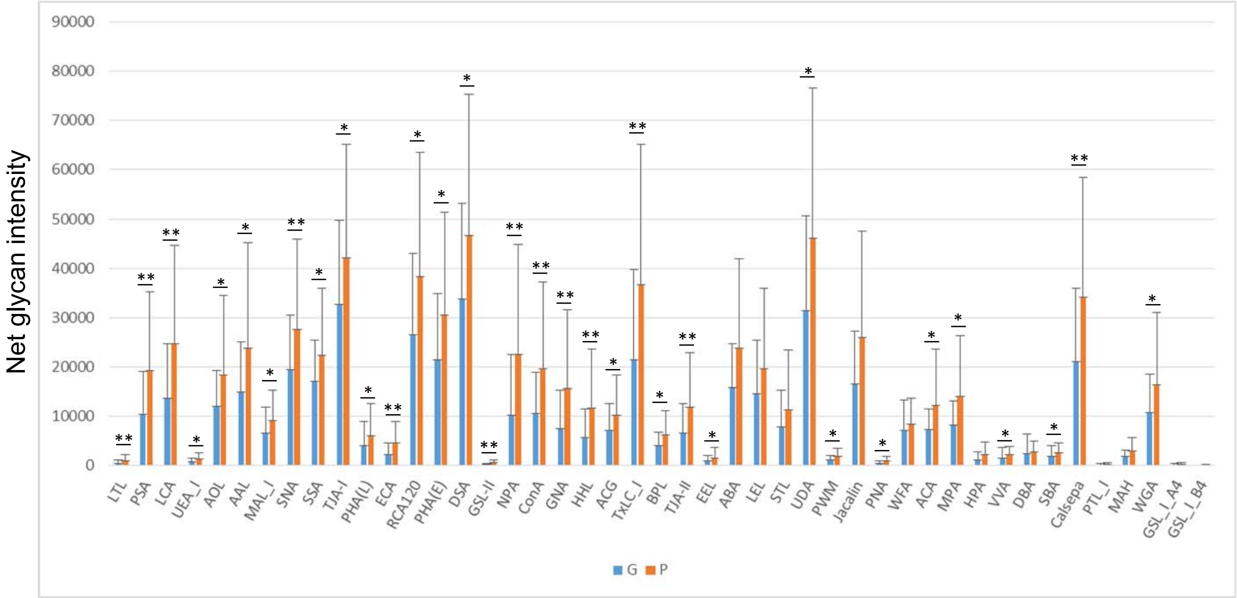

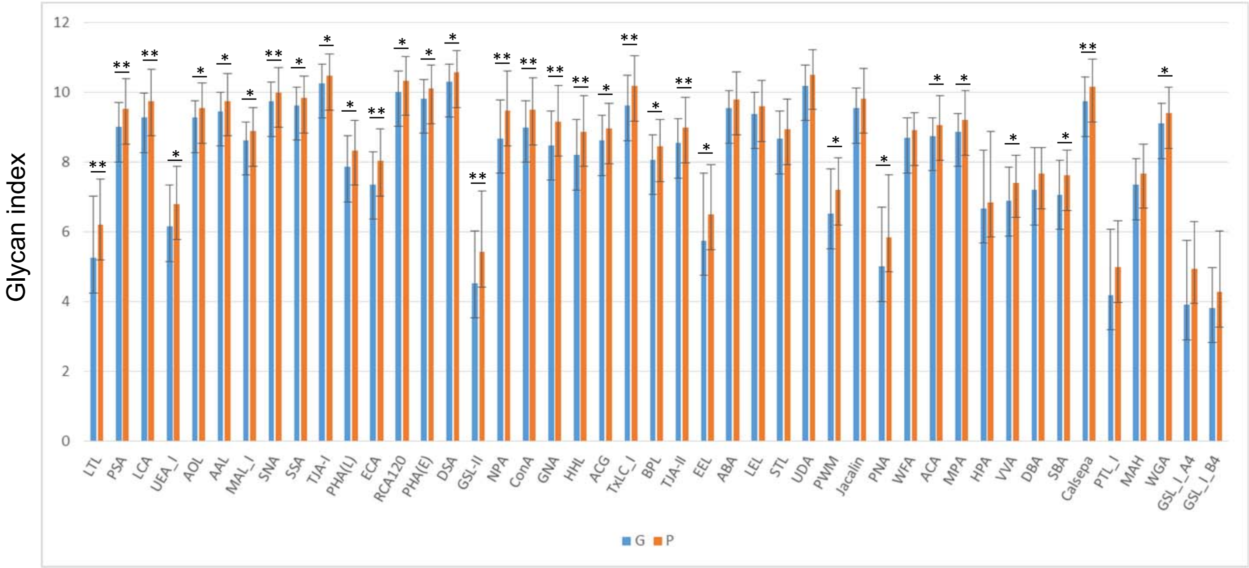

Supplementary Figure 2. Lectin binding signals in the patients with good prognosis (G group) and poor prognosis (P group). The lectin binding signals were analyzed by GlycoStation Tools Pro Ver.1.5. The values of lectin binding signals (Net-I; net glycan intensity) and glycan index (logNet-I) are shown. The bar indicates the mean, and the whisker shows the standard deviation. **P*<0.05, ***P*<0.01. Student's *t*-test. (StataCorp. 2015. Stata Statistical Software: Release 14. College Station, TX: StataCorp LP.)

Supplementary Figure 3

Lectins	*r*	*P*-value
LTL	0.4118	<0.0001*
PSA	0.7881	<0.0001*
LCA	0.7981	<0.0001*
UEA_I	0.4320	<0.0001*
AOL	0.6651	<0.0001*
AAL	0.7099	<0.0001*
MAL_I	0.5719	<0.0001*
SNA	0.6907	<0.0001*
SSA	0.6570	<0.0001*
TJAI	0.7337	<0.0001*
PHAL	0.5596	<0.0001*
ECA	0.5343	<0.0001*
RCA120	0.8036	<0.0001*
PHAE	0.7437	<0.0001*
DSA	0.6878	<0.0001*
GSLII	0.5326	<0.0001*
NPA	0.7638	<0.0001*
ConA	0.8314	<0.0001*
GNA	0.7707	<0.0001*
HHL	0.7678	<0.0001*
ACG	0.4329	<0.0001*
TxLC-I	0.8520	<0.0001*
BPL	0.6253	<0.0001*
TJAII	0.6913	<0.0001*
EEL	0.2580	0.0020
ABA	0.5076	<0.0001*
LEL	0.4447	<0.0001*
STL	0.4492	<0.0001*
UDA	0.7374	<0.0001*
PWM	0.6891	<0.0001*
Jacalin	0.5644	<0.0001*
PNA	0.4961	<0.0001*
WFA	0.5078	<0.0001*
ACA	0.5378	<0.0001*
MPA	0.5187	<0.0001*
HPA	0.3109	0.0002*
VVA	0.4231	<0.0001*
DBA	0.2782	0.0008*
SBA	0.4400	<0.0001*
Calsepa	0.8609	<0.0001*
PTL_I	0.3735	<0.0001*
MAH	0.3563	<0.0001*
WGA	0.5302	<0.0001*
GSL_I_A4	0.1368	0.1058
GSL_I_B4	0.0058	0.9457

$0.9 \le r < 1.0$
$0.8 \le r < 0.9$
$0.7 \le r < 0.8$
$0.6 \le r < 0.7$

Supplementary Figure 3. Simple correlation between lectin binding signals (net glycan intensity; Net-I) and urinary protein concentration. The r and P values are shown. The lectin binding signals were analyzed by GlycoStation Tools Pro Ver.1.5. Pearson correlation performed by Bonferroni correction. P<0.0011 is considered as significant (*). (StataCorp. 2015. Stata Statistical Software: Release 14. College Station, TX: StataCorp LP.)

Supplementary Figure 4

	LTL	PSA	LCA	UEA_I	AOL	AAL	MAL_I	SNA	SSA	TJAI	PHAL	ECA	RCA120	PHAE	DSA	GSLII	NPA	ConA	GNA	HHL	ACG	TxLC_I	BPL	TJAII	EEL	ABA	LEL	STL	UDA	PWM	Jacalin	PNA	WFA	ACA	MPA	HPA	VVA	DBA	SBA	Calsepa	PTL_I	MAH	WGA	GSL_I_A4	GSL_I_B4
LTL																																													
PSA	0.5102																																												
LCA	0.4888	0.9857																																											
UEA_I	0.6547	0.5251	0.5037																																										
AOL	0.4779	0.8929	0.909	0.5233																																									
AAL	0.4565	0.9097	0.9181	0.478	0.9403																																								
MAL_I	0.4681	0.8413	0.8566	0.5091	0.8597	0.8602																																							
SNA	0.3833	0.839	0.8844	0.4232	0.8674	0.8878	0.8475																																						
SSA	0.3712	0.7993	0.8542	0.4153	0.8429	0.8596	0.8484	0.9824																																					
TJAI	0.406	0.8689	0.9064	0.4253	0.8591	0.8922	0.8595	0.968	0.958																																				
PHAL	0.5117	0.8298	0.7858	0.5757	0.7691	0.7746	0.8291	0.6437	0.595	0.6865																																			
ECA	0.674	0.6625	0.6419	0.8258	0.6016	0.5997	0.5801	0.5403	0.5224	0.5515	0.6703																																		
RCA120	0.4079	0.9261	0.9546	0.4418	0.8854	0.9165	0.8576	0.9032	0.8867	0.9376	0.7441	0.5644																																	
PHAE	0.4379	0.9343	0.9454	0.4933	0.9243	0.9497	0.8988	0.8799	0.8552	0.9044	0.8313	0.6025	0.9596																																
DSA	0.3866	0.8915	0.9017	0.4389	0.8711	0.9177	0.9034	0.8556	0.8302	0.8923	0.8328	0.5435	0.923	0.9606																															
GSLII	0.5627	0.5735	0.5629	0.5999	0.5281	0.5803	0.5516	0.5121	0.4886	0.5218	0.527	0.5703	0.5206	0.555	0.546																														
NPA	0.4957	0.9221	0.9098	0.525	0.8384	0.8846	0.7863	0.7764	0.736	0.8121	0.7939	0.5781	0.898	0.9083	0.8722	0.5817																													
ConA	0.4809	0.9375	0.9483	0.496	0.8736	0.8685	0.81	0.8293	0.8101	0.8585	0.7386	0.616	0.9186	0.9037	0.8565	0.5515	0.8995																												
GNA	0.5076	0.912	0.8954	0.537	0.8389	0.8642	0.7603	0.7461	0.7076	0.782	0.7734	0.5824	0.8741	0.8836	0.8338	0.5643	0.9702	0.9208																											
HHL	0.4947	0.9134	0.8843	0.5414	0.812	0.8445	0.7436	0.7077	0.6643	0.7505	0.7949	0.61	0.8552	0.8669	0.8241	0.5576	0.9544	0.9091	0.9829																										
ACG	0.3567	0.6762	0.7193	0.3115	0.7098	0.6905	0.8511	0.7438	0.7495	0.7633	0.684	0.3933	0.7359	0.7413	0.784	0.395	0.644	0.6791	0.6133	0.5903																									
TxLC_I	0.442	0.9431	0.9447	0.4879	0.842	0.8853	0.8007	0.8138	0.7793	0.8736	0.7912	0.5815	0.9568	0.9351	0.9031	0.5476	0.9332	0.9177	0.9093	0.9016	0.6653																								
BPL	0.5358	0.7767	0.7322	0.6254	0.7738	0.7672	0.6488	0.5529	0.5148	0.5777	0.7663	0.6545	0.6877	0.7768	0.711	0.5171	0.8125	0.7514	0.8342	0.8291	0.4291	0.7607																							
TJAII	0.4712	0.899	0.9078	0.5	0.904	0.9411	0.8957	0.8736	0.8415	0.9027	0.8372	0.5829	0.927	0.9479	0.9327	0.5871	0.9073	0.8643	0.8793	0.8539	0.7854	0.9105	0.7424																						
EEL	0.3668	0.3264	0.3379	0.3848	0.345	0.3985	0.387	0.3542	0.3508	0.357	0.3782	0.3737	0.3402	0.367	0.372	0.4328	0.3637	0.313	0.3288	0.283	0.3262	0.332	0.3854	0.4428																					
ABA	0.3297	0.6825	0.7634	0.3619	0.7816	0.7444	0.7903	0.8827	0.908	0.8503	0.4361	0.4155	0.7824	0.7442	0.7176	0.4262	0.6233	0.7148	0.5924	0.5324	0.7402	0.6459	0.3918	0.7424	0.3303																				
LEL	0.2943	0.6575	0.7185	0.3669	0.7703	0.8052	0.842	0.8527	0.8724	0.833	0.5659	0.4217	0.8021	0.8097	0.8068	0.4414	0.6823	0.6633	0.6372	0.5879	0.7791	0.6727	0.4549	0.821	0.3412	0.8608																			
STL	0.3755	0.6219	0.6642	0.4499	0.7167	0.7815	0.7954	0.761	0.7705	0.7579	0.5662	0.3989	0.7522	0.7774	0.7709	0.4985	0.729	0.6273	0.6911	0.634	0.7241	0.67	0.5425	0.8138	0.4374	0.7517	0.9272																		
UDA	0.4217	0.9037	0.9219	0.4494	0.8615	0.9119	0.8832	0.8867	0.8695	0.9312	0.7583	0.5361	0.9622	0.9439	0.9431	0.5627	0.9073	0.8857	0.8696	0.8571	0.7824	0.9263	0.6606	0.9394	0.352	0.7836	0.837	0.8122																	
PWM	0.5475	0.6845	0.6522	0.6614	0.5914	0.6018	0.4889	0.4797	0.449	0.5113	0.5766	0.6564	0.5909	0.6278	0.5406	0.6084	0.7094	0.6952	0.7284	0.7504	0.3101	0.6689	0.7307	0.5743	0.2223	0.3499	0.3454	0.4234	0.5606																
Jacalin	0.3332	0.7375	0.8095	0.3648	0.8123	0.7989	0.8271	0.9212	0.9432	0.9022	0.5129	0.4375	0.8513	0.8053	0.7811	0.4273	0.6796	0.754	0.6486	0.5954	0.7749	0.7192	0.429	0.8051	0.354	0.9777	0.8972	0.7895	0.8461	0.3732															
PNA	0.6128	0.5637	0.5601	0.6106	0.5635	0.5427	0.5454	0.4826	0.4679	0.4996	0.5712	0.4992	0.5137	0.5593	0.5404	0.6524	0.5706	0.5502	0.5601	0.5252	0.4667	0.5602	0.5818	0.5959	0.4843	0.4244	0.3884	0.4684	0.5184	0.5922	0.4484														
WFA	0.4638	0.7565	0.7076	0.4495	0.7441	0.7221	0.7439	0.5189	0.4865	0.5743	0.886	0.5727	0.6659	0.7773	0.751	0.4393	0.7208	0.699	0.7428	0.7562	0.5765	0.7183	0.8475	0.7491	0.3057	0.3466	0.4603	0.4763	0.6546	0.5639	0.406	0.5036													
ACA	0.4118	0.7419	0.7945	0.4382	0.8213	0.8107	0.8437	0.8837	0.8911	0.8564	0.5437	0.4567	0.8058	0.799	0.7825	0.5149	0.7218	0.7592	0.6962	0.6383	0.7698	0.707	0.5197	0.8192	0.3874	0.9568	0.8765	0.829	0.8373	0.4295	0.9444	0.5057	0.4462												
MPA	0.3659	0.7452	0.8076	0.413	0.8384	0.803	0.8508	0.9052	0.9143	0.8739	0.5609	0.4638	0.8119	0.7925	0.7769	0.4638	0.6939	0.7534	0.6698	0.6163	0.7958	0.6977	0.4828	0.8193	0.3815	0.9694	0.8669	0.7794	0.8217	0.383	0.9684	0.4816	0.4661	0.9614											
HPA	0.2509	0.2837	0.2529	0.4681	0.2609	0.3661	0.2774	0.233	0.2174	0.2441	0.3198	0.2248	0.3016	0.3361	0.3127	0.4229	0.4651	0.2275	0.4213	0.3957	0.1554	0.3802	0.4616	0.4007	0.3434	0.1247	0.2935	0.5093	0.3483	0.4222	0.1828	0.4407	0.252	0.2813	0.1997										
VVA	0.6167	0.593	0.5572	0.7741	0.5978	0.5786	0.6334	0.4275	0.4071	0.4539	0.7687	0.7367	0.5001	0.6039	0.5702	0.5537	0.6191	0.5603	0.6251	0.6164	0.4885	0.5686	0.7911	0.6444	0.4846	0.3242	0.4148	0.5144	0.5161	0.6331	0.3483	0.6608	0.752	0.4417	0.4327	0.4771									
DBA	0.511	0.4459	0.4076	0.6278	0.4372	0.4305	0.53	0.2995	0.282	0.3335	0.6863	0.6455	0.3763	0.4721	0.4432	0.4451	0.4454	0.3811	0.4331	0.4498	0.4302	0.4344	0.6086	0.5037	0.3551	0.196	0.3302	0.3965	0.4001	0.4546	0.2315	0.4811	0.6764	0.294	0.2933	0.4286	0.8592								
SBA	0.548	0.5768	0.5154	0.7182	0.5529	0.5277	0.5196	0.3252	0.2827	0.3567	0.7778	0.7206	0.4499	0.566	0.5184	0.5012	0.607	0.5306	0.6161	0.6372	0.338	0.5492	0.8367	0.5715	0.4168	0.1449	0.2604	0.3567	0.4409	0.6671	0.1861	0.5766	0.7871	0.269	0.263	0.4388	0.9128	0.7645							
Calsepa	0.4458	0.9312	0.9583	0.4793	0.8624	0.8683	0.8103	0.8706	0.8577	0.9078	0.6973	0.5792	0.9553	0.9075	0.8626	0.552	0.8808	0.9596	0.8835	0.8654	0.6996	0.9423	0.6814	0.872	0.3133	0.7623	0.6996	0.6562	0.9165	0.6488	0.8102	0.5654	0.6321	0.7798	0.788	0.2493	0.5117	0.3508	0.4555						
PTL_I	0.3702	0.3668	0.381	0.2676	0.4325	0.4746	0.3952	0.3933	0.3997	0.3998	0.3318	0.2993	0.3731	0.424	0.3929	0.4148	0.3666	0.3963	0.3806	0.3219	0.3318	0.3718	0.4068	0.4783	0.442	0.3831	0.3542	0.4273	0.3734	0.401	0.3879	0.5645	0.3753	0.4405	0.413	0.2786	0.457	0.3346	0.3403	0.3998					
MAH	0.4576	0.5602	0.6098	0.546	0.6554	0.645	0.7695	0.6712	0.7042	0.6607	0.5306	0.4531	0.6205	0.6224	0.6308	0.4878	0.606	0.5886	0.5927	0.5303	0.7408	0.5464	0.469	0.7143	0.5003	0.7721	0.7526	0.7949	0.6661	0.3555	0.7616	0.5552	0.4196	0.8292	0.818	0.3652	0.606	0.4379	0.3888	0.61	0.4777				
WGA	0.4146	0.7884	0.8155	0.4645	0.8404	0.8736	0.9203	0.8599	0.8536	0.8589	0.7499	0.5134	0.8559	0.8837	0.8818	0.52	0.8061	0.7547	0.7549	0.7159	0.8343	0.7942	0.6327	0.9203	0.4158	0.8111	0.9199	0.9056	0.8991	0.4477	0.8533	0.5166	0.643	0.8853	0.8615	0.412	0.5904	0.4852	0.4656	0.7657	0.4271	0.7851			
GSL_I_A4	0.3579	0.1675	0.1852	0.25	0.2463	0.1683	0.1966	0.1741	0.1728	0.1471	0.1348	0.3243	0.1159	0.1383	0.1229	0.3147	0.1179	0.2869	0.1789	0.1503	0.1977	0.1151	0.2557	0.1999	0.1956	0.2339	0.0821	0.0793	0.1021	0.2501	0.1582	0.3507	0.2213	0.2487	0.2378	-0.1281	0.3079	0.1963	0.2405	0.214	0.381	0.2284	0.1457		
GSL_I_B4	0.0363	-0.0098	-0.0139	0.0269	0.0711	0.0277	0.0137	0.0011	-0.0069	-0.0155	0.0038	-0.054	-0.0197	0.0073	0.0312	0.0235	0.0447	0.036	0.0377	-0.0135	0.0089	0.0015	0.1459	0.0582	0.2255	0.0498	-0.0396	0.0231	-0.0254	0.0341	0.0179	0.1884	0.0855	0.0851	0.0619	0.0254	0.1655	0.0816	0.1036	-0.0221	0.2405	0.1122	0.0301	0.2863	

- Fucose
- Sialyc acid
- Acialo-
- Agalacto-
- High-mannose branch
- Galctose
- Chitin
- O-Glycan
- Others

- r＝1.000
- 0.9≦ r <1.000
- 0.8≦ r <0.9
- 0.7≦ r <0.8
- 0.6≦ r <0.7

Supplementary Figure 4. Correlation matrix among 45 lectin binding signals. The lectin binding signals were analyzed by GlycoStation Tools Pro Ver.1.5 and the r values are shown. (StataCorp. 2015. Stata Statistical Software: Release 14. College Station, TX: StataCorp LP.)

Supplementary Figure 5

Lectins	*r*	*P*-value
LTL	0.1459	0.0833
PSA	0.2778	0.0008*
LCA	0.3255	0.0001*
UEA_I	0.1915	0.0224
AOL	0.3121	0.0002*
AAL	0.285	0.0006*
MAL_I	0.3104	0.0002*
SNA	0.3865	<0.0001*
SSA	0.4039	<0.0001*
TJAI	0.3536	<0.0001*
PHAL	0.1415	0.0931
ECA	0.1321	0.117
RCA	0.3388	<0.0001*
PHAE	0.3055	0.0002*
DSA	0.2755	0.0009*
GSLII	0.2105	0.0119
NPA	0.2569	0.002
ConA	0.3564	<0.0001*
GNA	0.2724	0.001
HHL	0.2561	0.0021
ACG	0.3704	<0.0001*
TxLC-I	0.2664	0.0014
BPL	0.0467	0.581
TJAII	0.3214	0.0001*
EEL	0.1641	0.051
ABA	0.4872	<0.0001*
LEL	0.3803	<0.0001*
STL	0.333	0.0001*
UDA	0.3725	<0.0001*
PWM	0.1291	0.1258
Jacalin	0.4701	<0.0001*
PNA	0.2336	0.0052
WFA	0.0285	0.7364
ACA	0.4921	<0.0001*
MPA	0.4589	<0.0001*
HPA	0.0857	0.3104
VVA	0.0682	0.42
DBA	0.0046	0.957
SBA	-0.0512	0.5449
Calsepa	0.3844	<0.0001*
PTL_I	0.2148	0.0103
MAH	0.3872	<0.0001*
WGA	0.3443	<0.0001*
GSL_I_A4	0.2086	0.0127
GSL_I_B4	-0.1284	0.1278

$0.9 \leq r < 1.0$
$0.8 \leq r < 0.9$
$0.7 \leq r < 0.8$
$0.6 \leq r < 0.7$

Supplementary Figure 5. Simple correlation between lectin binding signals and age. The r and P values are shown. The lectin binding signals were analyzed by GlycoStation Tools Pro Ver.1.5. Pearson correlation performed by Bonferroni correction. P<0.0011 is considered as significant (*). (StataCorp. 2015. Stata Statistical Software: Release 14. College Station, TX: StataCorp LP.)

Supplementary Figure 6

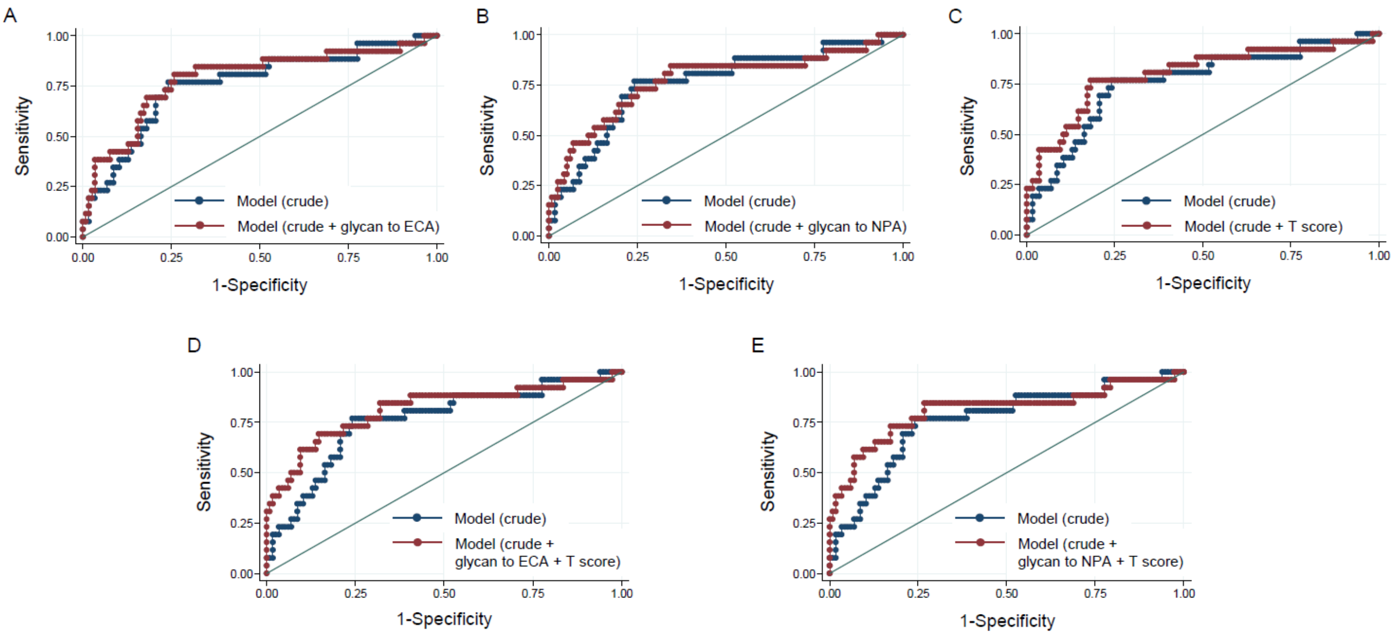

Supplementary Figure 6. Receiver operating curves (ROC) of the estimation models with and without glycan index (ECA and NPA) and T score. Covariates (crude) were age, sex, estimated glomerular filtration rate, and log-transformed urinary protein excretion at the time of renal biopsy. (StataCorp. 2015. Stata Statistical Software: Release 14. College Station, TX: StataCorp LP.)

Supplementary Figure 7

a

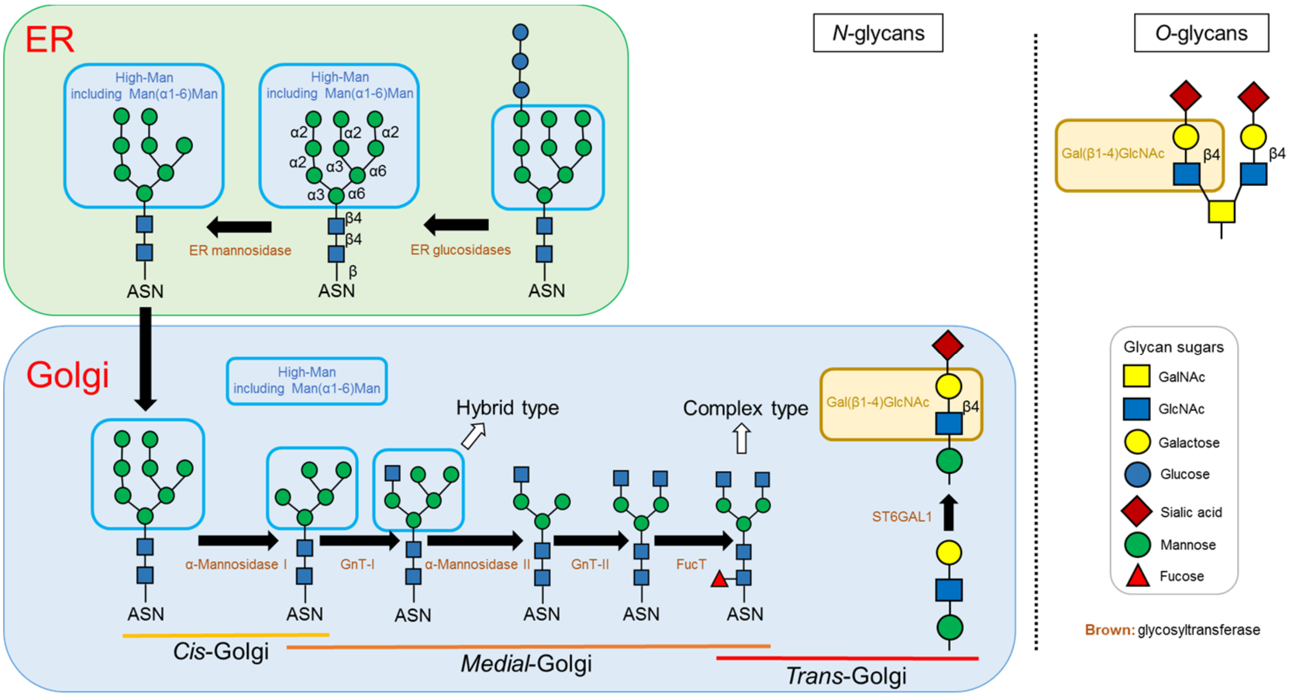

b

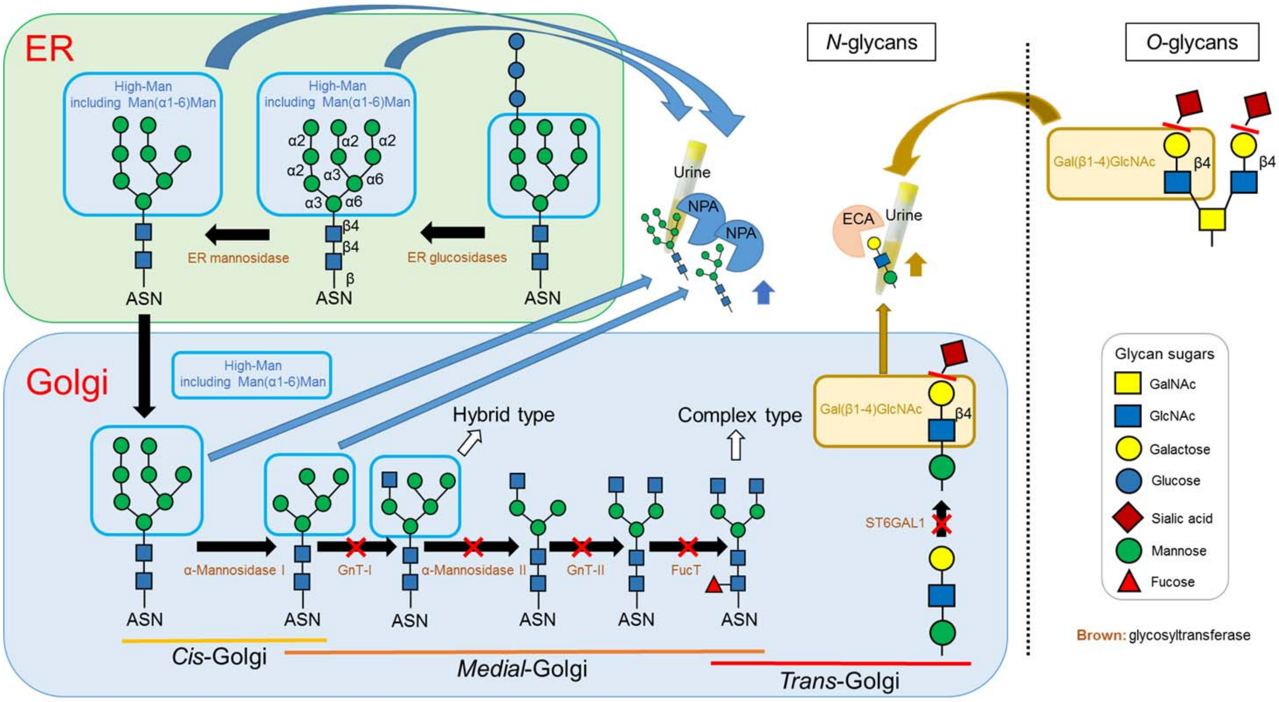

Supplementary Figure 7. Mechanism of glycosylation and the putative relationships between synthesis of glycans and urinary glycan excretion.

a. Schematic illustration of the biosynthesis and processing of N-glycans and the glycosylation processes required to create mature N-glycans. Processing of N-glycans begins in the ER. Following transfer of Glc3Man9GlcNAc2 (Glc: glucose) to protein, three Glc residues and a Man residue are removed by ER glucosidases and ER mannosidase respectively. High-mannose type N-glycans are transported to the Golgi. Trimming of the α1-2Man residue is performed by α-Mannosidase I in the Cis-Golgi. In the Medial-Golgi, the addition of the first GlcNAc residue by N-acetylglucosaminyltransferase (GnT-I) generates GlcNAcMan5GlcNAc2 and α-Mannosidase II trims two Man residues to form the GlcNAcMan3GlcNAc2 precursor of complex N-glycans. The second GlcNAc is added by the action of GnT-II. Moreover, N-glycan matures with further sugar additions in the Trans-Golgi, such as the addition of α1-6Fucose to the asparagine (Asn)-linked GlcNAc in the N-glycan core. In addition, it is known that sialyltransferases mostly functions in the Trans-Golgi.

b. Mechanisms for increased urinary excretions of recognized glycans. In IgA nephropathy, glycosylation abnormalities in the Trans-Golgi and Medial-Golgi may decrease normal glycans and increase the urinary excretion of immature glycan components and terminal epitopes, such as high-mannose including Man(α1-6)Man (recognized by NPA) and Gal(β1-4)GlcNAc (recognized by ECA). Gray fonts indicate glycosyltransferases. Abbreviations: ER, endoplasmic reticulum; ECA, Erythrina cristagalli; NPA, Narcissus pseudonarcissus; GnT, acetylglucosaminyltrasferase; FucT, fucosyltransferase.
